# Transcriptional regulation of root development in cacao: a genome-wide comparative analysis between zygotic and somatic embryo-derived seedlings

**DOI:** 10.3389/fpls.2026.1816111

**Published:** 2026-06-03

**Authors:** Rolande Eugenie Ango Makondy, Julien Agneessens, Wenbin Wei, Nicolas Niemenak, Keith Lindsey, Alexandre Mboene Noah

**Affiliations:** 1 Department of Biological Science, Higher Teachers’ Training College, University of Yaounde´ I, Yaounde, Cameroon; 2 Department of Biosciences, Durham University, Durham, United Kingdom; 3 Department of Biochemistry, Faculty of Science, University of Douala, Douala, Cameroon

**Keywords:** abiotic stress, RNA sequencing, root development, somatic embryogenesis, *Theobroma cacao*, transcription factors, vascular patterning

## Abstract

**Introduction:**

The knowledge gaps on cacao root biology deeply hamper the possibility to efficiently develop tissue culture-based regeneration adapted to cacao physiology. Dissecting the regulatory mechanisms controlling cacao root development and identifying molecular disorders associated with root defects in somatic embryo-derived seedlings (SES) is critical to elucidate potential targets for optimizing somatic embryogenesis protocols as well as to develop resilient elite genotypes that can tolerate environmental alterations. The current study aimed to systematically identify the transcriptional patterns underlying cacao root development in zygotic embryo-derived seedlings (ZES) and SES, with a focus on the emergence of lateral roots.

**Methods:**

The study examined roots from four cacao seedling types: 4−day−old ZES, 7−day−old ZES, a lateral−rootless SES variant, and SES with fully developed lateral roots. Root morphology was assessed through tissue microscopy, and whole−root transcriptomes were profiled using genome−wide RNA sequencing (RNA−seq).

**Results:**

Histological analysis revealed mispatterning of pericycle and vascular tissues in roots of lateral-rootless SES. Genome−wide transcriptomic profiling revealed 12,979 differentially expressed genes, encompassing pathways related to amino acid and carbohydrate metabolism, phytohormone signaling, mitogen-activated protein kinase (MAPK) cascades, motor−protein function, and homologous recombination—core regulatory modules that underpin cacao root patterning and adaptive plasticity. More than 900 transcription factors, including members of the AP2/ERF, MYB, NAC, LOB, GNAT, SET, and WRKY families, were also differentially regulated. Our work identified common and distinctive features of root growth and lateral branching in ZES and in normal and misshaped SES at the transcriptional level.

**Discussion:**

Collectively, our results show that early root morphogenesis in cacao is orchestrated by precise transcriptional reprogramming. Yet, the heightened stress associated with *in vitro* culture perturbs this developmental trajectory in SES, inducing pericycle and vascular tissue defects that ultimately constrain primary root growth and lateral root initiation.

## 1 Introduction

*Theobroma cacao* L. (cacao) is a perennial crop of the *Malvaceae* family. It stands as a linchpin of tropical agro-economies and is cultivated primarily for its beans, which underpin the global chocolate industry. The unique flavor and aroma of cacao beans, combined with their rich nutritional composition, have established cacao as both a cultural staple and a focus of extensive scientific research. Cacao and its derivatives are replete with a spectrum of bioactive compounds, including methylxanthines (theobromine, theophylline, and caffeine) and a diverse array of polyphenols, encompassing flavanols, procyanidins, anthocyanins, and phenolic acids (Bertazzo et al., 2013; Febrianto and Zhu, 2022). Given its multifaceted significance and rising demand, sustaining and enhancing cacao productivity amid climate change necessitates developing elite genotypes that combine high yield with robust biotic and abiotic stress resistance. However, cacao’s allogamous reproductive system complicates the fixation and propagation of these desirable traits through conventional breeding. In this context, somatic embryogenesis (SE) has emerged as a transformative platform, affording unparalleled opportunities for the clonal propagation, genetic improvement, and dissemination of superior cacao lines (Maximova et al., 2002; Li et al., 1998). SE is particularly valued for its scalability and its capacity to preserve the genetic integrity of elite germplasm, thereby circumventing the limitations inherent to sexual reproduction.

Despite the several advantages of SE, its practical deployment in cacao improvement is challenged by inefficiencies at the embryo-to-seedling conversion stage, with functional root system establishment representing a critical bottleneck. Yet, root system architecture, which determines seedling vigor, nutrient uptake, and adaptation to environmental fluctuations, is crucial for successful acclimatization and subsequent field performance. Adjustments of culture media, including sucrose supplementation and osmotic modulation, have shown some efficacy in promoting somatic embryo conversion (Valencia-Lozano et al., 2021). Nevertheless, the physiological and molecular mechanisms underlying these responses remain poorly characterized. These molecular insights are critical for dissecting the cacao plant’s responses to abiotic stress and for pinpointing candidate genes that can be targeted through molecular breeding or genetic engineering.

As a dicotyledonous plant, the cacao root system consists of the primary root formed during embryogenesis and lateral roots that are formed post-embryonically (Lavenus et al., 2013). Development of the primary root is driven by the root apical meristem, which gives rise to different root tissues and supports continuous elongation (Jiang and Feldman, 2005; Aichinger et al., 2012). Lateral roots, by contrast, originate *de novo* from xylem pole pericycle cells of the primary root through a five-step process: (i) positioning in the oscillation zone, (ii) lateral root initiation via nuclear migration in specified founder cells up to the first asymmetric division, (iii) lateral root primordium organogenesis, (iv) lateral root emergence through overlying tissues, and (v) vascular connection and meristem activation. The four latter developmental stages take place in the differentiation root zone (Laskowski and Ten Tusscher, 2017; Banda et al., 2019). All these processes are tightly controlled by an intricate interplay of gene regulatory networks and plant hormones, particularly auxin, which acts as a master regulator (Du and Scheres, 2018; Motte et al., 2019). Hormonal crosstalk further refines root development by coordinating cell division, elongation, and stress adaptation (Liu et al., 2014). Most importantly, regulation of gene expression constitutes the central mechanism that activates and coordinates the diverse regulatory pathways governing root growth and lateral branching. Gene expression is regulated at the transcriptional level predominantly by transcription factors (TFs) and post-transcriptional events (Zhao et al., 2016; Kumar and Mohapatra, 2021). While the regulatory machinery associated with root development is well-characterized in model plants like *Arabidopsis* (Beckers et al., 2025; Moore et al., 2025), the molecular basis of root development in cacao, especially in the context of SE, remains largely unexplored. Although prior studies in cacao reported hormonal profiling in root at early seedling development (Noah et al., 2021), no genome−wide RNA sequencing (RNA−seq) studies have yet dissected the transcriptional programs that shape this critical organ.

To address this gap, we conducted a comparative transcriptomic analysis and hypothesized that, in cacao, relative to zygotic embryo−derived seedlings (ZES), the developmental anomalies observed in somatic embryo−derived seedlings (SES)—particularly root malformation—arise from disrupted metabolic processes and misregulation of key developmental regulators during *in vitro* culture. We integrated root morpho−anatomical characterization with high−throughput transcriptomics to capture the global dynamics of transcriptome reprogramming during early post−germination in ZES and SES. This approach enabled the construction of a comprehensive transcriptomic atlas of cacao roots, defining the molecular architecture of root development. The dataset further revealed regulatory defects underlying SES−associated root anomalies, a major limitation in cacao SE. By resolving the pathways and candidate regulators that govern root patterning and functional performance, this work establishes the foundation for enhancing root vigor in *in vitro*−derived seedlings and informs next−generation genetic engineering strategies aimed at producing high−yielding, climate−resilient cacao genotypes essential for sustaining global chocolate supply chains.

## 2 Materials and methods

### 2.1 Plant materials and growing conditions

The present study was performed with the cacao genotype “SCA6” (Scavina 6), which is included in the gene bank of the Institute of Agricultural Research for Development at Nkolbisson (Yaounde, Cameroon). Both ZES and SES were used as biological materials. Fresh seeds were harvested from mature cacao pods. After removal of the seed coat, they were surface sterilized by immersion in 3% (v/v) sodium hypochlorite solution and then rinsed three times in sterile water (10 min for each rinse). Seeds were sown in jars containing half-strength DKW medium (Driver and Kuniyuki, 1984) under sterile conditions and incubated in a growth chamber in darkness at 25 ± 1 C. ZES derived from these seeds were sampled at defined developmental stages.

Secondary somatic embryos were produced according to a protocol adapted from Minyaka et al. (2008) and Maximova et al. (2002). The derived embryos were cultured on embryo germination medium for 60 days to induce radicle protrusion and early seedling development. The embryo germination medium was composed of the DKW mineral complex (Driver and Kuniyuki, 1984) and 90 g L^−1^ anhydrous sucrose. Cultures were maintained in the dark at 25 ± 1 C. To achieve homogeneity between germination starting materials, only vigorous and well-shaped cotyledonary somatic embryos (2–2.5 cm long) were selected. Morphological observations were conducted to document the early seedling establishment of cacao zygotic and somatic embryos. Primary root length and number of lateral roots were recorded from 1 to 10 days post-germination for ZES and after 60 days of culture for SES. Three replicates were carried out with 25 seedlings in each replicate.

### 2.2 Plant tissue sample collection

ZES were sampled at two phenological stages—BBCH07 and BBCH08—corresponding to 4 and 7 days after the initiation of germination (DAI). This timeframe was selected because it spans the window of lateral root initiation and emergence in cacao (Niemenak et al., 2010). SES were codified based on their root architecture as compared to the ZES: SES-BBCH07-like (abbreviated as SEBBCH07Lik) referred to lateral-rootless SES and SES-BBCH08-like (abbreviated as SEBBCH08Lik) referred to SES displaying both primary and lateral roots. SEBBCH07Lik and SEBBCH08Lik are not developmental stages but rather same−age SES with distinctive root morphotypes. SEBBCH07Lik displays developmental arrest and cannot progress to SEBBCH08Lik. In total, there were four seedling types analyzed throughout this experiment: ZEBBCH07 (ZES at 4 DAI, exhibiting only tap roots), ZEBBCH08 (ZES at 7 DAI, with primary plus lateral roots), SEBBCH07Lik, and SEBBCH08Lik.

### 2.3 Paraffin sectioning of cacao root seedlings

Primary roots of ZES and SES (five per seedling type) were randomly collected, and the root differentiation zone was sliced in segments of 4mm from the shootward part. Tissues were fixed for 48h in a fixative solution containing 2.5% glutaraldehyde (v/v) and 3% paraformaldehyde (w/v), pH = 7.2. After fixation, the samples were dehydrated by moving through a graded series of ethanol (30%, 50%, 70%, 95%, and 100%) for an hour each, cleared in xylene for 3h and finally infiltrated and embedded with paraffin wax. Transverse and longitudinal serial sections up to 14 µm thickness were subsequently obtained on a manual rotary microtome. Once sections were cut, they were floated on a warm water bath that helped remove wrinkles. Afterwards, the sections were stretched and adhered in microscope glass slides with a plate heated up to 40 C. After dewaxing, tissues were rehydrated and stained with a 0.02% Toluidine blue (O’Brien et al., 1964) for 1min, and air dried. Stained sections were then examined and photographed with a Zeiss Axioskop compound optical microscope (Carl Zeiss, Cambridge, UK), equipped with a QImaging Retiga-2000r camera (Photometrics, Marlow, UK). In addition, the diameter of the vascular cylinder and the number of vessels were determined from images using the Cell counter tool in the ImageJ software (https://imagej.net/ij/index.html).

### 2.4 RNA sequencing

#### 2.4.1 Total RNA isolation and quantification

The entire root was sampled from each seedling category in triplicate, each consisting of a poolof at least three plants. Once harvested, root samples were immediately transferred to a solution ofLysis Buffer A (LBA) + 1-Thioglycerol (TG) buffer to ensure rapid lysis and inactivation of nucleases. Samples were stored at −80°C or directly processed for total RNA isolation using the ReliaPrepTM RNA tissue Miniprep System Kit (Promega, USA). RNA concentration, purity, and integrity were checked using an Agilent 5400 Analyzer (Aligent Technologies, Santa Clara, CA, USA; **Supplementary Table 1**).

#### 2.4.2 Library construction, sequencing, and read mapping

Messenger RNA was purified from total RNA using poly-T oligo-attached magnetic beads. After fragmentation, the first-strand cDNA was synthesized using random hexamer primers, followed by the second-strand cDNA synthesis using dTTP for non-directional library construction (Parkhomchuk et al., 2009). Libraries were ready after end repair, A-tailing, adapter ligation, size selection, amplification, and purification. Libraries were checked with a Qubit fluorometer (Thermo Fisher Scientific, UK) for size distribution detection and real-time PCR for quantification. Quantified libraries were pooled and sequenced on an Illumina platform, according to effective library concentration and data amount. RNA-seq was carried out using Illumina NovaSeq 6000 by Novogene (Cambridge, UK). Raw sequencing data were obtained from the Illumina platform in FASTQ format. These reads were subsequently filtered to remove adapters, poly-N sequences, and low-quality reads. Concurrently, quality metrics including Q20, Q30, and GC content were calculated to ensure data integrity. High-quality clean reads were aligned to the *T. cacao* Matina 1-6 (v1.1) reference genome (Motamayor et al., 2013) using HISAT2 v2.0.5 (Kim et al., 2019). The Matina cultivar, a Forastero type, was selected as the reference due to its high genomic synteny with Scavina 6 (an Upper Amazon Forastero clone). Mapped reads for each sample were assembled using StringTie v1.3.3b (Pertea et al., 2015) following a reference-based approach to identify both known and novel transcripts. Functional annotation was performed against multiple databases, including Pfam, Swiss-Prot, Gene Ontology (GO), and Kyoto Encyclopedia of Genes and Genomes (KEGG).

### 2.5 Data analysis

#### 2.5.1 Quantification of gene expression level

To assess the reproducibility and reliability of data to reality, gene expression measurements were performed across technical and biological replicates. Each sample was quantified for gene expression levels using the featureCounts v1.5.0-p3 software (Liao et al., 2014); expression matrices were combined to obtain read counts, which were converted to FPKM (Fragments Per Kilobase of transcript sequence per Millions base pairs sequenced). To compare gene expression levels under different conditions, the distribution of gene expression levels and FPKM among different samples was displayed by boxplots.

#### 2.5.2 Sample correlation analysis

Pearson’s correlation coefficient was used to measure the degree of correlation between samples. The value of *r*^2^ ranges from 0 to 1, and the closer the *r*^2^ value is to 1, the higher the degree of correlation. Simultaneously, the PCA of gene expression was plotted to assess data repetition of samples.

#### 2.5.3 Differential expression analysis and functional enrichment

Differential expression analysis was performed using read counts and the DESeq2 R/package v1.20.0 (Varet et al., 2016). The resulting *p*-values were adjusted using the Benjamini and Hochberg’s approach for controlling the false discovery rate. The selection criteria for DEGs were |log2(Fold Change)| ≥ 1 and adjusted *p*-value (*p*_adj_) ≤ 0.05. The co-expression Venn diagram, hierarchical clustering, and Volcano maps of the DEGs were plotted and analyzed. In addition, GO and KEGG enrichment analysis were performed to identify biological functions or pathways associated with DEGs (Kanehisa and Goto, 2000). Functional enrichment was done using GO and KEGG database resources (http://www.geneontology.org/ and http://www.genome.jp/kegg/). The enrichment analysis was conducted with cluster Profiler R package, and gene length bias was corrected as well (Yu et al., 2012). GO and KEGG terms with *p*_adj_ < 0.05 were considered significant enrichment. Pairwise differential expression analysis was conducted between ZES and SES intra- and inter-groups, i.e., ZEBBCH07 vs. ZEBBCH08, SEBBCH07Lik vs. SEBBCH08Lik, ZEBBCH07 vs. SEBBCH07Lik, and ZEBBCH8 vs. SEBBCH08Lik. Prediction of TF families was performed by using BLASTX similarity from the Plant Transcription Factor Database (PlantTFDB v5.0; https://planttfdb.gao-lab.org/) with the default parameters.

#### 2.5.4 Gene co-expression analysis

To move from single-gene list to systems-level insight, gene co-expression analysis was conducted on our 12 different tissue samples using the K-means clustering algorithm on the iDEP 0.96 web application (Ge et al., 2018). Gene expression was normalized using *Z*-score normalization. The GO biological process (BP) and KEGG enrichment for the resulting clusters were performed.

#### 2.5.5 Statistical analysis methods

One-way analysis of variance (ANOVA), followed by *post-hoc* multiple comparison using the Tukey method, *p* ≤ 0.05, was conducted. Graphs were generated using the R 4.5.1 software, and final images were prepared in Inkscape vector graphics editor.

## 3 Results

### 3.1 Ontogenetic features of early seedling development of zygotic and somatic embryos of *Theobroma cacao*

**Figure 1:**
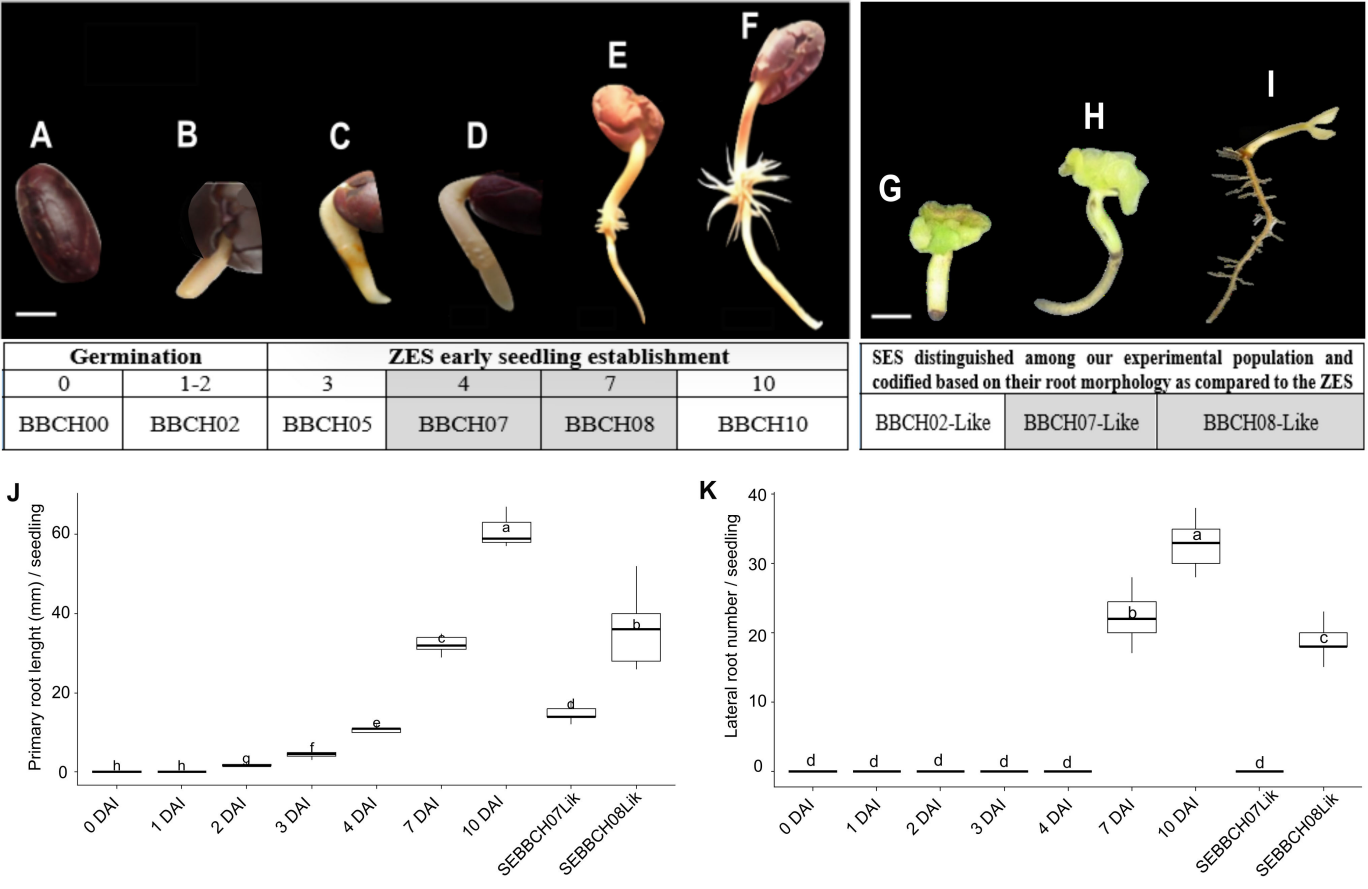
Root morphology of Theobroma cacao during early seedling growth. Developing ZES at **(A)** 0, **(B)** 1–2, **(C)** 3, **(D)** 4, **(E)** 7, and **(F)** 10 days after initiation of germination (DAI). **(G)** Rootless SES; **(H)** lateral rootless SES; **(I)** SES bearing both primary and lateral roots. **(J)** Primary root length of cacao seedlings. **(K)** Number of lateral roots. Scale bars represent 1 cm **(A–F)** and 0.5 cm **(G–I)**. The 0, 1–2, 3, 4, 7, and 10 DAI stages correspond to BBCH00, BBCH02, BBCH05, BBCH07, BBCH08, and BBCH10, respectively, according to the BBCH scale codification (Niemenak et al., 2010). Seedling morphotypes highlighted in grey indicate the four types analyzed for transcriptome profiling. SEBBCH07Lik and SEBBCH08Lik are not developmental stages but same age SES with distinct root morphotypes. Letters on boxplots indicate statistical significance; identical letters denote no significant differences.

Cacao seedling morphogenesis was monitored over 10 days for ZES and 60 days for SES. Developmental milestones were codified according to the extended BBCH scale (Niemenak et al., 2010), establishing a standardized comparative framework for both systems. Early ZES development (**Figure 1**) was dominated by root and hypocotyl emergence. Zygotic embryos (**Figure 1A**) initiated germination within 24 h post-imbibition, and radicle protrusion occurred approximately 2 DAI, corresponding to scale point BBCH02 (**Figure 1B**), followed by rapid elongation and hypocotyl extension. By 3 DAI (BBCH05; **Figure 1C**), root hairs differentiated. At 4 DAI (BBCH07), an apical hook became evident; the elongation of radicle had typically produced a primary root with length reaching ~1 cm long (**Figure 1D**). At 7 DAI (BBCH08), lateral roots emerged while the primary root extended to ~3 cm (**Figure 1E**). By 10 DAI (BBCH10), the lateral root system became denser whereas the primary root lengthened and enlarged continuously (**Figures 1F, J, K**). Hypocotyl elongation occurred during the transition from 4 to 7 DAI. Throughout the experimental timeframe, cotyledons remained closed and the plumule had not yet emerged, indicating a developmental prioritization of root and hypocotyl morphogenesis over shoot expansion. Overall, developing ZES exhibit remarkable morphological uniformity and a high plant conversion rate (~98%).

In contrast, germination and early growth of SES were slow, asynchronous, and often stunted, precluding precise temporal characterization of developmental milestones. Early SES growth showed cotyledons turning light green and opening to reveal the plumule. Hypocotyl elongation was typically limited, resulting in shorter and slimmer seedlings compared to ZES. SES often exhibited a striking morphological variability, root architecture being the most discriminating feature observed. Three categories of SES could be distinguished based on their root morphology: the rootless SES (13.34% ± 2.76%; **Figure 1G**), the lateral-rootless SES (SEBBCH07Lik) with only primary root (41.33% ± 3.05%; **Figure 1H**), and finally those displaying a more normal root system with both primary and lateral roots (SEBBCH08Lik), representing the most advanced and vigorous phenotype (45.33% ± 4.27%; **Figure 1I**). SES lacking roots showed dwarfism and stunted growth. Lateral-rootless SES showed intermediate characteristics with continued elongation of primary root without branching. In contrast, propagates with a well-developed root system were robust and showed no signs of growth deficiency, exhibiting a complex root architecture extending from primary to tertiary roots. The next sections will only focus on ZEBBCH07, ZEBBCH08, SEBBCH07Lik, and SEBBCH08Lik, the four seedling types considered for histological and transcriptomic analyses.

### 3.2 Pericycle and vascular mispatterning impairs root development in cacao somatic embryo seedlings

**Figure 2:**
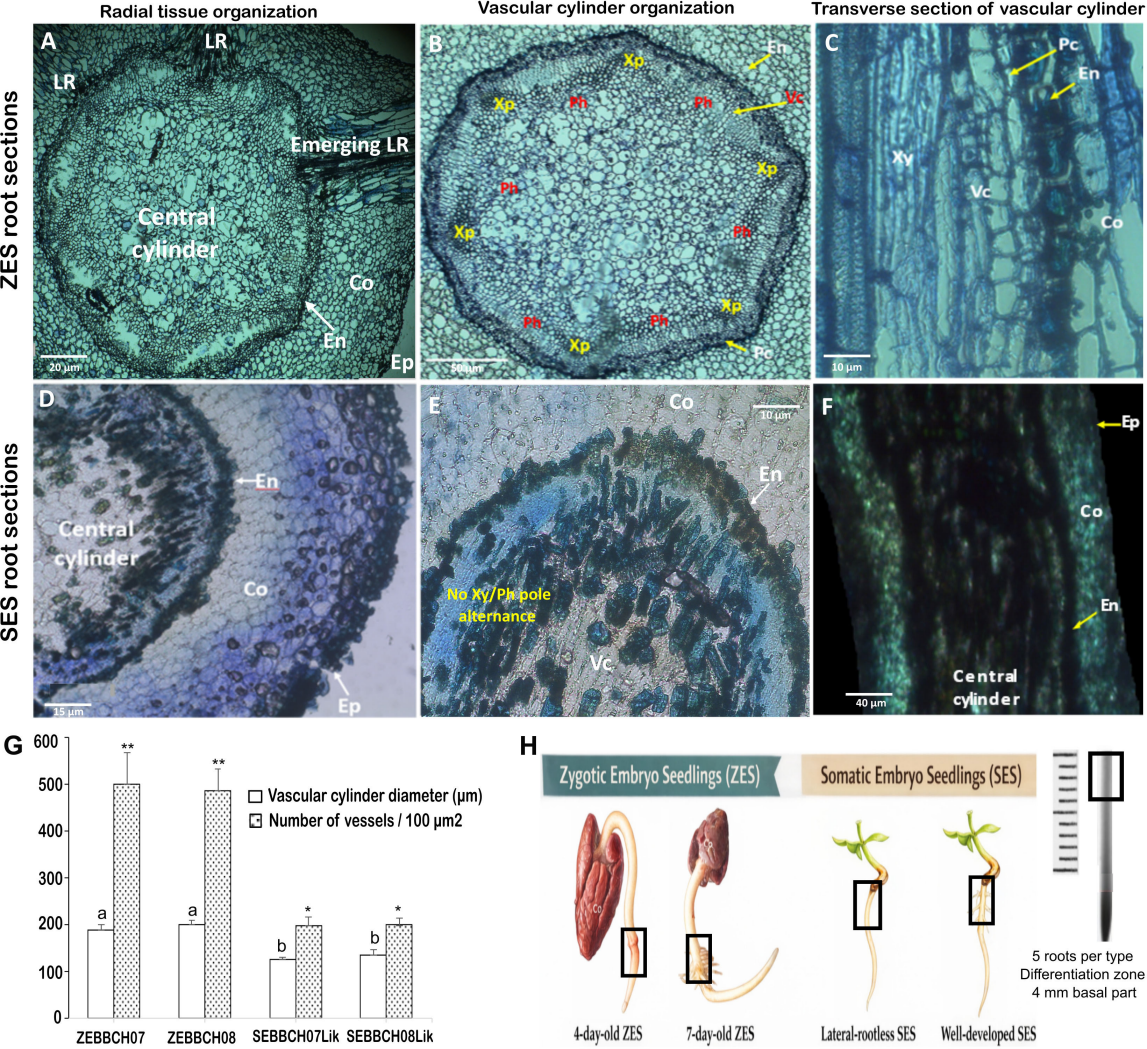
Histological sections of cacao young primary root at the differentiation zone. Transverse section of primary root of **(A)** ZES and **(D)** SES. Transverse section showing the central vascular cylinder organization of **(B)** ZES and **(E)** SES. Vascular cylinder of longitudinal section of **(C)** ZEBBCH07/08 and **(F)** SEBBCHLik07. Co, Cortex; En, endodermis; Ep, epidermis; Pc, pericycle; Ph, phloem; Vc, vascular tissue; Xy, xylem. **(G)** Diameter of vascular cylinders and number of vessels per 100 µm^2^ in the vascular cylinder. (H) Experimental design of the tissue microscopy experiment. Letters on barplots represent statistical significances; the same letters/asterisks denote no significant differences.

Histological examination was conducted on root tissues focusing on the differentiation zone (**Figure 2H**), to elucidate structural disparities between the four seedling types: ZEBBCH07, ZEBBCH08, SEBBCH07Lik, and SEBBCH08Lik. In ZEBBCH07, lateral root primordia at different developmental stages were observed (**Figure 2A**). However, lateral root emergence did not occur in ZEBBCH07 but was evident in ZEBBCH08. In ZES, epidermis, cortex, endodermis, pericycle, and central vascular cylinder were arranged in a complete and correctly organized manner (**Figures 2A–C**). The central cylinder exhibited a canonical organization of six alternating xylem and phloem poles (hexarch structure), indicative of advanced vascular differentiation (**Figure 2B**). Although SES displayed a superficially normal radial tissue arrangement (**Figure 2D**), root tissues were less structurally complete with disrupted xylem/phloem poles and misaligned pericycle cells compared to ZES. The oversized vascular cylinder contained many vessel strands randomly distributed (**Figure 2E**). Moreover, lateral-rootless SES (SEBBCH07Lik) exhibit profound aberrations, reflecting impaired cellular patterning. Individual vessel elements were not fully elongated, were frequently interrupted, and were improperly aligned to each other (**Figure 2F**). In addition, ZES had a greater number of vessels than SES, and the diameter of the vascular cylinder was significantly larger in ZES (**Figure 2G**).

### 3.3 Functional annotation and pathway enrichment of root development-associated DEGs in zygotic and somatic embryo cacao seedlings

**Figure 3:**
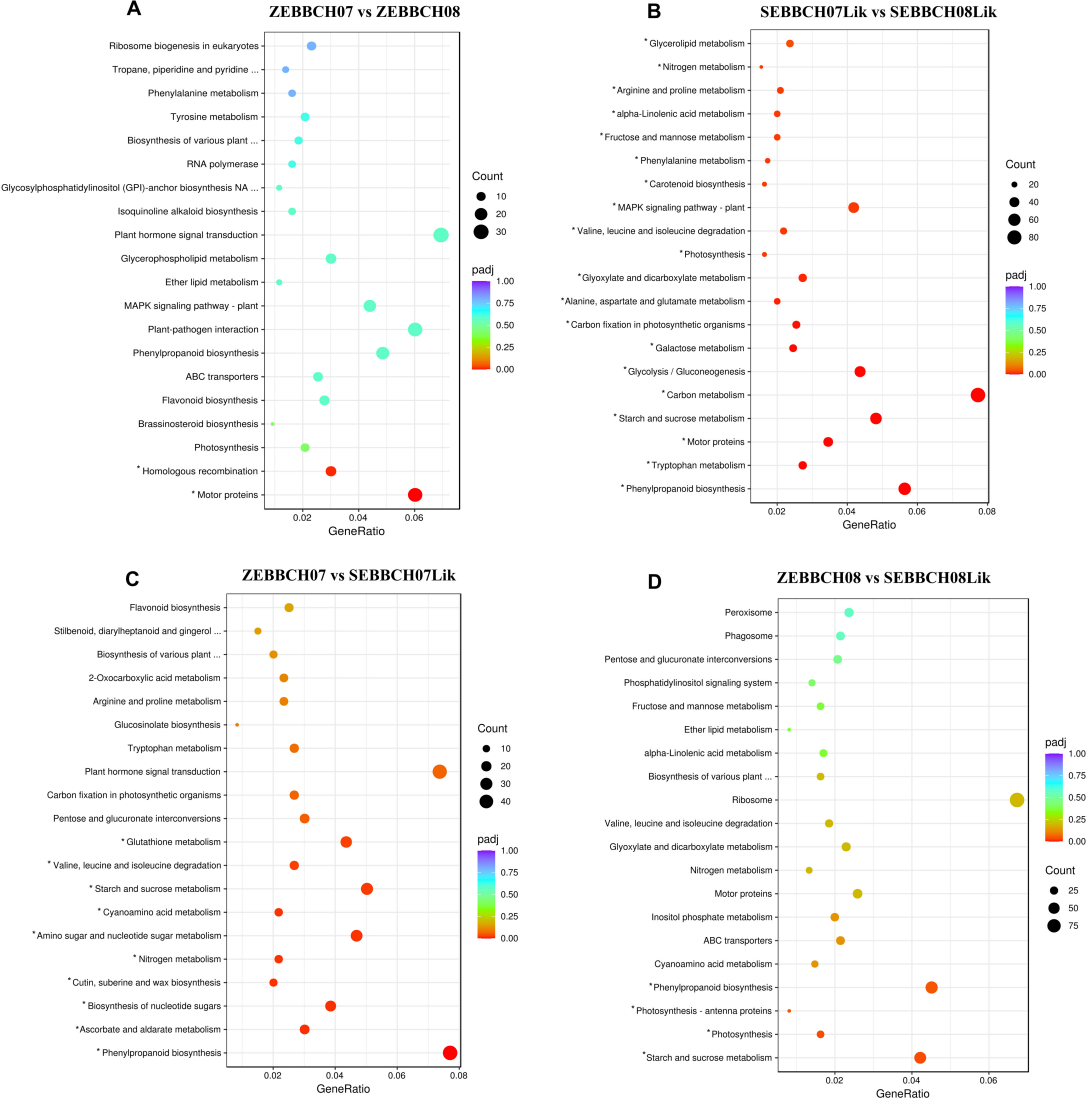
Gene ontology (GO) enrichment analysis of DEGs involved in the process of early root development in cacao. Overview of the top 10 GO terms belonging to “MF”, “CC”, and “BP” categories across **(A)** ZEBBCH07 vs. ZEBBCH08, **(B)** SEBBCH07Lik vs. SEBBCH08Lik, **(C)** ZEBBCH07 vs. SEBBCH07Lik, and **(D)** ZEBBCH08 vs. SEBBCH08Lik pairwise comparisons. The horizontal axis in the image represents the ratio of the number of DEGs annotated on the GO term to the total number of DEGs (GeneRatio), while the vertical axis represents the GO term. The size of the dots indicated the number of DEGs in this pathway. The dot colour indicates the adjusted p-value (padj); the smaller is the padj, the higher is the enrichment degree. BP, biological process; CC, cellular component; and MF, molecular functions. GO terms highlighted using “*” are significantly enriched.

To explore the molecular events associated with cacao root development, a high-resolution transcriptome analysis was performed on the entire root system of the four seedling types—ZEBBCH07, ZEBBCH08, SEBBCH07Lik, and SEBBCH08Lik—each with three biological replicates. Gene expression metrics revealed the high quality, suitability, and reliability of our RNA-seq data (**Supplementary Sheets Data S1**, **S2**). Pairwise differential expression analysis was conducted on raw counts with stringent thresholds (*p*_adj_ < 0.05 and |log2FoldChange| ≥ 1.0) using the DEseq2 software (Varet et al., 2016). To elucidate the putative biological roles of the DEGs, a comprehensive functional annotation was performed by mapping the DEGs to GO terms. GO enrichment analysis was rigorously implemented using *p*_adj_ <0.05 as significant cutoff. Based on these criteria, significant enrichment was observed across GO categories: BP, cellular component (CC), and molecular function (MF). Specifically, 3, 2, 5, and 6 BP terms; 6, 4, 0, and 4 CC terms; and 15, 23, 27, and 17 MF terms were significantly enriched in the ZEBBCH07 vs. ZEBBCH08, SEBBCH07Lik vs. SEBBCH08Lik, ZEBBCH07 vs. SEBBCH07Lik, and ZEBBCH08 vs. SEBBCH08Lik comparisons, respectively (**Supplementary Data Sheet S3**—Sheets A, B, C, and D). The top 10 enriched terms within each GO category are provided in **Figure 3**. The ZEBBCH07 vs. ZEBBCH08 comparison was predominantly enriched in “binding”, “catalytic activity”, and “motor activity” terms. In addition, the BP category had the most DEGs involved in the “response to stimulus”, “cellular response to DNA damage stimulus”, “response to stress”, “microtubule-based movement and process”, and “DNA repair” (**Figure 3A**; **Supplementary Data Sheet S3**—Sheet A). Meanwhile, in the SEBBCH07Lik vs. SEBBCH08Lik group, the most enriched GO terms in the MF category were “transcription regulator activity”, “DNA binding transcription regulator activity”, and “heme binding”. While the BP GO terms included “transmembrane transport”, “carbohydrate metabolic process”, “response to stimulus”, and “response to oxidative stress” (**Figure 3B**; **Supplementary Data Sheet S3**—Sheet B). When examining ZES/SES pairwise combinations, GO terms such as “transporter activity”, “catalytic activity”, “response to oxidative stress”, and “cellular carbohydrate metabolic process” were highly enriched in ZEBBCH07 vs. SEBBCH07Lik, whereas “carbohydrate metabolic process”, “transmembrane transport”, and “catalytic activity” predominated in ZEBBCH08 vs. SEBBCH08Lik (**Figures 3C, D**; **Supplementary Data Sheet S3**—Sheets C and D). Despite some overlap in enriched GO terms across the comparative groups, notable variations were observed in the specific gene constituents and their relative expression levels (**Supplementary Figure 1**).

**Figure 4:**
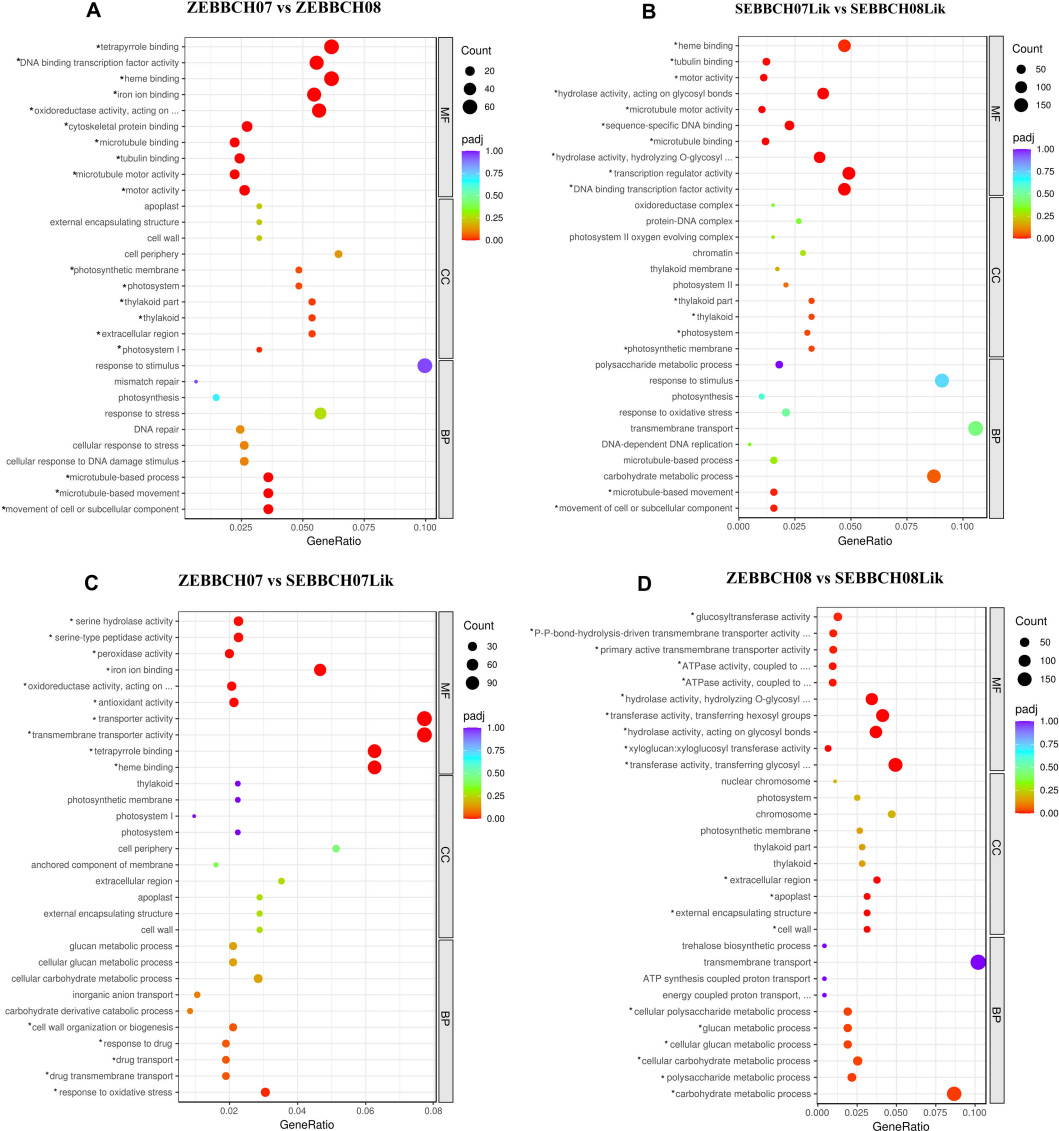
KEGG enrichment analysis of DEGs involved in the process of early cacao root development. Overview of the top 20 KEGG pathways enriched in **(A)** ZEBBCH07 vs. ZEBBCH08, **(B)** SEBBCH07Lik vs. SEBBCH08Lik, **(C)** ZEBBCH07 vs. SEBBCH07Lik, and **(D)** ZEBBCH08 vs. SEBBCH08Lik pairwise comparisons. The x-axis indicates the Gene ratio showing the degree of KEGG pathway enrichment. The y-axis indicates the name of the KEGG pathway. The size of the dots indicated the number of DEGs in this pathway. The dot colour indicates the adjusted p-value (padj); the smaller is the padj, the higher is the enrichment degree. KEGG pathways highlighted using “*” are significantly enriched.

To further delineate the biological pathways in which the identified DEGs may participate during *T. cacao* root growth and lateral root emergence, a pathway-centric analysis was performed using the KEGG database. A total of 114, 121, 116, and 121 KEGG pathways were found to be enriched in the ZEBBCH07 vs. ZEBBCH08, SEBBCH07Lik vs. SEBBCH08Lik, ZEBBCH07 vs. SEBBCH07Lik, and ZEBBCH08 vs. SEBBCH08Lik groups, respectively, among which 2, 22, 10, and 4 pathways reached statistical significance at *p*_adj_ < 0.05 (**Supplementary Data Sheet S4**—Sheets A, B, C, and D). The top 20 KEGG pathways with the highest DEG representation are illustrated in **Figure 4**. In the ZEBBCH07 vs. ZEBBCH08 comparison, the most prominent KEGG pathways included “motor proteins”, “homologous recombination”, “plant hormone signal transduction”, and “plant–pathogen interaction” (**Figure 4A**; **Supplementary Data Sheet S4**—Sheet A). Of the 22 KEGG pathways significantly enriched in the SEBBCH07Lik vs. SEBBCH08Lik group, the most enriched were “carbon metabolism”, “phenylpropanoid biosynthesis”, “starch and sucrose metabolism”, and “MAPK signalling pathway” (**Figure 4B**; **Supplementary Data Sheet S4**—Sheet B). Comparative analyses between ZES and SES combinations revealed that “phenylpropanoid biosynthesis”, “plant hormone signal transduction”, “starch and sucrose metabolism”, “amino sugar and nucleotide sugar metabolism”, and “glutathione metabolism” were predominantly enriched in ZEBBCH07 vs. SEBBCH07Lik (**Figure 4C**; **Supplementary Data Sheet S4**—Sheet C). In the ZEBBCH08 vs. SEBBCH08Lik group, “starch and sucrose metabolism”, “phenylpropanoid biosynthesis”, “motor proteins”, “glyoxylate and dicarboxylate metabolism”, and “ABC transporters” were most prominent (**Figure 4D**; **Supplementary Data Sheet S4**—Sheet D).

In parallel, co-expression analysis using K-means clustering applied to the most variable 2,000 expressed genes (standard derivation > 0.25) and partitioned the DEG dataset into six distinct expression clusters (**Supplementary Figure 3**). Cluster 1 exhibited a transient upregulation specifically in lateral-rootless SES. Cluster 1 functional enrichment analysis showed GO BP terms associated with lipid storage, seed oil body biogenesis, and chemical homeostasis, which may indicate activation of reserve accumulation pathways typical of quiescent or stress-adapted states. Clusters enriched in nitrogen and amino acid metabolism, mitogen-activated protein kinase (MAPK) signaling, reactive oxygen species (ROS), and hydrogen peroxide metabolism (clusters 2, 4, and 5) showed strong induction in well-developed SES roots and reduced expression in lateral-rootless SES. Genes preferentially expressed in ZES were enriched in cell wall biogenesis, and motor proteins (Clusters 3, 4, and 6).

Alongside TF candidates, KEGG enrichment analysis revealed motor−protein-related processes, carbohydrate, amino acid and nitrogen metabolism, glutathione metabolism, phenylpropanoid biosynthesis, plant hormone signal transduction, MAPK signaling, and epigenetic and homologous recombination pathways as key regulatory modules of root development in *T. cacao*. These pathways were therefore prioritized for subsequent in−depth functional analyses.

### 3.4 Cytoskeletal dynamics and wall remodelling jointly reflect impaired cellular patterning in somatic embryo-derived seedling roots

**Figure 5:**
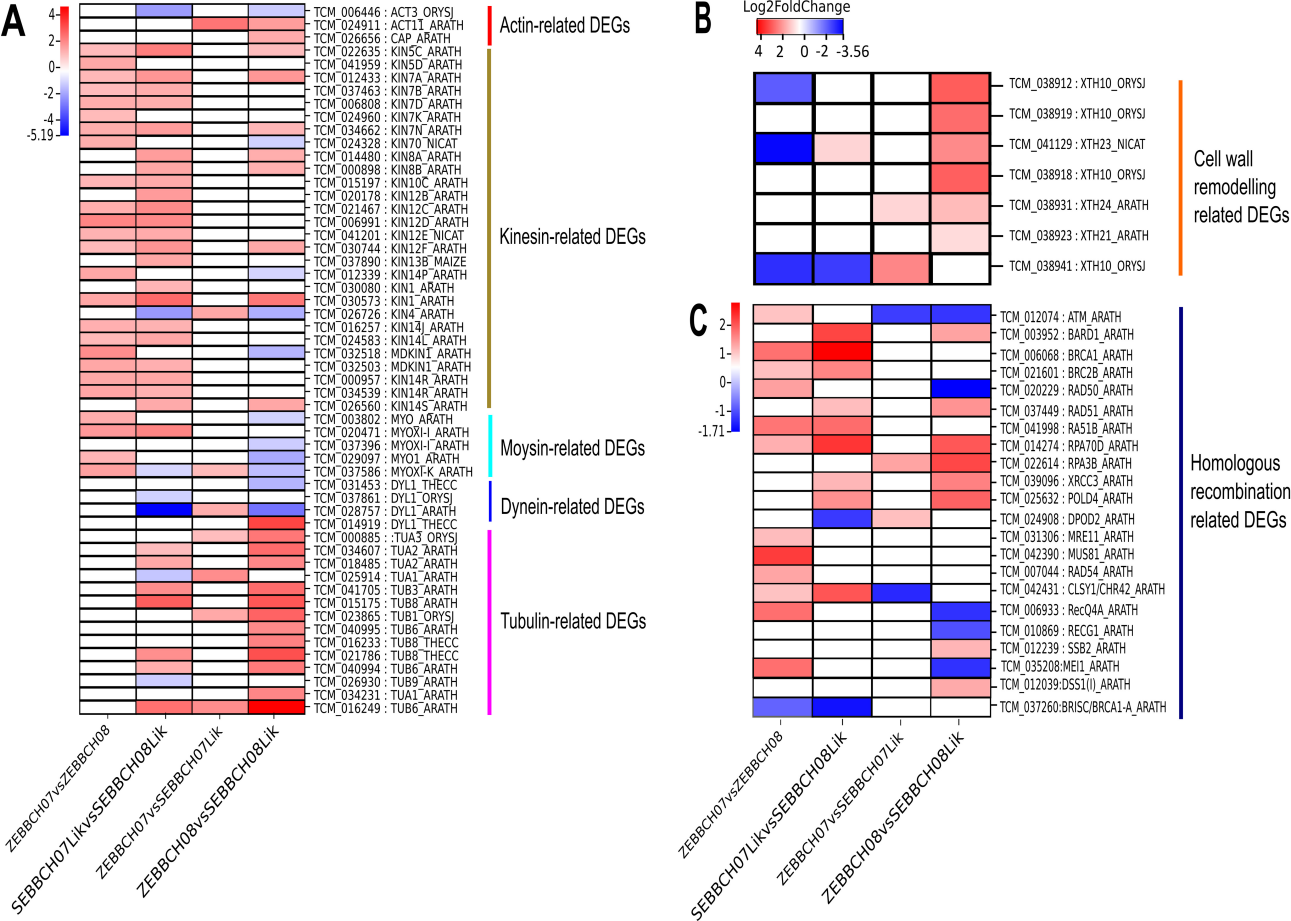
Analysis of DEGs associated to motor proteins, cell wall remodelling, and homologous recombination in the process of early cacao root development. Heatmaps of DEGs associated with **(A)** motor proteins, **(B)** cell wall remodeling, and **(C)** homologous recombination in ZEBBCH07 vs. ZEBBCH08, SEBBCH07Lik vs. SEBBCH08Lik, ZEBBCH07 vs. SEBBCH07Lik, and ZEBBCH08 vs. SEBBCH08Lik pairwise comparison groups.

The transcriptomic landscape of motor proteins revealed differential expression patterns that mirror substantial implication in the cellular patterning during root development. KEGG enrichment analysis revealed that the “motor protein” term was significantly enriched in both ZES and SES intra-group comparisons (*p*_adj_ < 0.05; **Supplementary Data Sheet S4**—Sheets A and B). In total, we identified 55 DEGs related to “motor protein” including 28 kinesins, 5 myosins, 3 actins, 5 dyneins, and 14 tubulins (**Figure 5A**; **Supplementary Figure 2A**; **Supplementary Data Sheet S5**—Sheet A). Comparative analyses between ZES and SES showed that motor-protein-encoding DEGs were more highly expressed in ZES (**Figure 5A**), aligning with their robust root formation. In contrast, SES exhibited upregulation of microtubule depolymerizer kinesin-13 (TCM_037890: *TcKIN13B_MAIZE*), particularly in lateral root-deficient morphotype, which might perturb cortical microtubule arrays and compromise xylem differentiation. Regarding cell-wall remodeling, seven cacao xyloglucan endotransglucosylase/hydrolase (XTH) genes—homologs of *OsXTH10*, *NaXTH23*, and *AtXTH21*/24—were differentially expressed and showed context-dependent regulation: they were downregulated in the ZEBBCH07 vs. ZEBBCH08 and SEBBCH07Lik vs. SEBBCH08Lik comparisons, but upregulated in ZES versus SES (**Figure 5B**; **Supplementary Figure 2B**; **Supplementary Data S5**—Sheet A). These patterns point to dynamic cell-wall remodeling in the branched-rooted morphotype and in the ZES versus SES comparison.

### 3.5 Metabolic adaptation in somatic embryo seedlings drives root development under *in vitro* stress conditions

**Figure 6:**
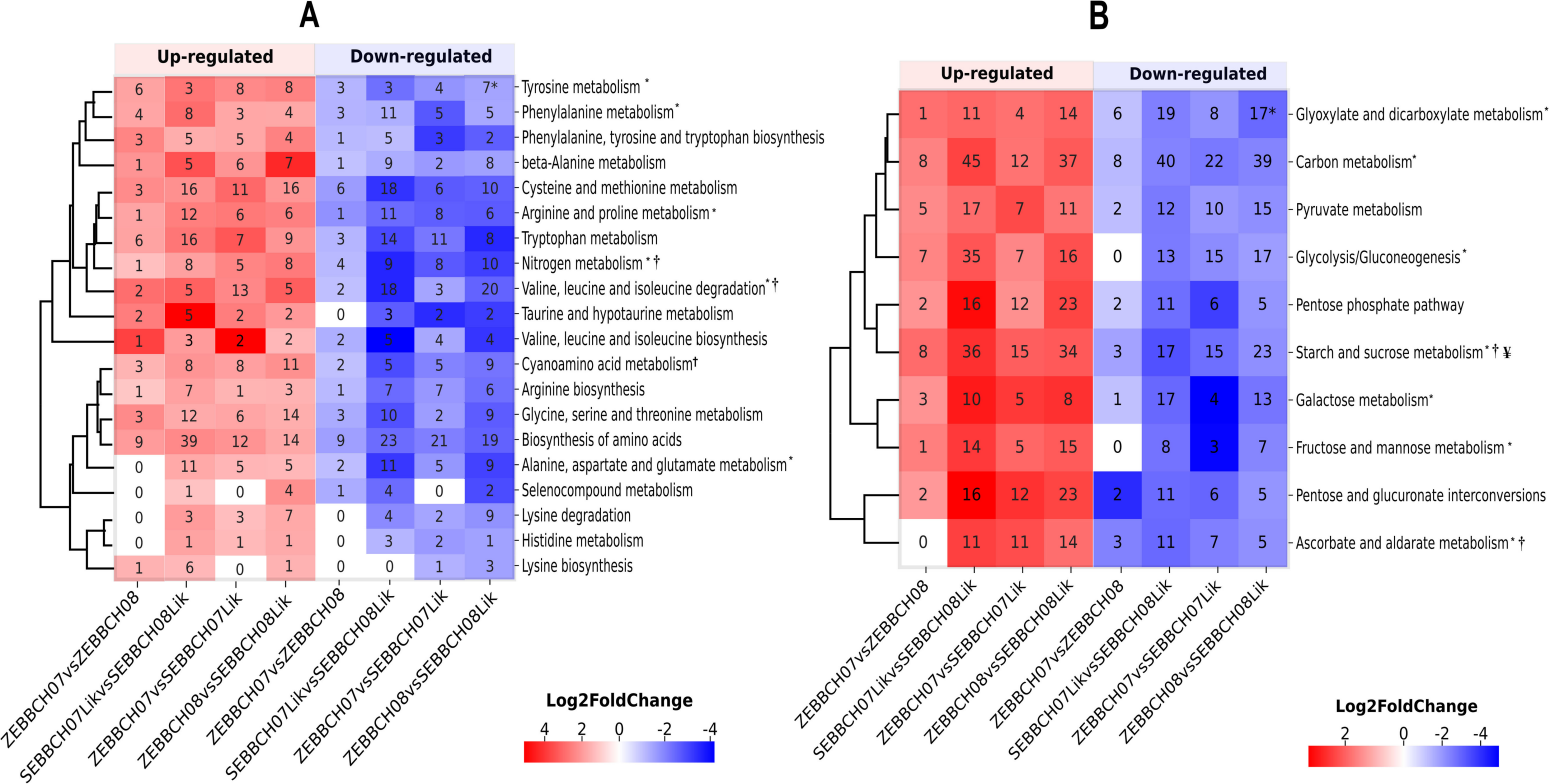
KEGG enrichment analysis of DEGs associated to amino acid, nitrogen, carbon and carbohydrate metabolisms in the process of early cacao root development. Heatmap analysis on the distribution (numbers and LogFold Changes) of up-and downregulated DEGs related to **(A)** aminoacid and nitrogen as well as **(B)** carbon and carbohydrate in ZEBBCH07 vs. ZEBBCH08, SEBBCH07Lik vs. SEBBCH08Lik, ZEBBCH07 vs. SEBBCH07Lik, and ZEBBCH08 vs. SEBBCH08Lik pairwise comparisons. Numbers in the heatmap represent the counts of up-and downregulated DEGs in each KEGG pathway while the color indicates the Log2FoldChanges. Pathways highlighted using “*”, “†”, and “¥” are significantly enriched in SEBBCH07Lik vs. SEBBCH08Lik, ZEBBCH07 vs. SEBBCH07Lik, and ZEBBCH08 vs. SEBBCH08Lik pairwise comparisons, respectively.

KEGG enrichment analysis revealed 271 DEGs and 28 DEGs involved in amino acid and nitrogen metabolism, respectively (**Supplementary Data Sheet S5**—Sheet B). In the ZEBBCH07 vs. ZEBBCH08 comparison, none of the amino acid or nitrogen metabolic pathways displayed statistically significant enrichment (*p*_adj_ < 0.05). However, the SEBBCH07Lik vs. SEBBCH08Lik group exhibited significant enrichment in six pathways, including “nitrogen metabolism”, “tryptophan metabolism”, “alanine, aspartate and glutamate metabolism”, “valine, leucine and isoleucine degradation”, “phenylalanine metabolism”, and “arginine and proline metabolism” (**Supplementary Data Sheet S4**—Sheets A and B). The distribution of these DEGs is visually represented in **Figure 6A**. Substantial disparities were observed in both the number and expression levels of DEGs across the experimental groups. SES demonstrated a pronounced increase in the number and stronger expression of DEGs related to nitrogen and amino acid metabolism compared to ZES, indicating a robust reprogramming of these pathways during SE root growth and lateral root branching in *T. cacao*. Pathways associated with tryptophan, cysteine and methionine, alanine, aspartate and glutamate, and branched-chain amino acids (valine, leucine, and isoleucine) were especially influenced in both SES/SES and ZES/SES comparisons. DEGs associated with these pathways were most highly expressed in SEBBCH08Lik seedlings, which exhibited pronounced lateral root branching, suggesting that these metabolic routes may drive lateral root development during cacao SE. Conversely, taurine and hypotaurine DEGs were upregulated in SEBBCH07Lik seedlings lacking lateral branching, aligning with previous reports indicating that these amino acids, especially hypotaurine, negatively impact lateral root formation (Fang et al., 2014; **Figure 6A**).

Sugar metabolism, critical for plant development, also showed significant modulation. A total of 119 and 286 DEGs—across nine pathways—were enriched in carbon and carbohydrate metabolism, respectively (**Supplementary Data Sheet S5**—Sheet C). The distribution and intensity of up- and downregulated DEGs associated with carbon and carbohydrate metabolism across our four pairwise sample comparisons are provided in **Figure 6B**. The heatmap analysis clearly showed that SES, especially those with lateral root branching, have a higher number and stronger expression of DEGs in these metabolic pathways compared to ZES. Moreover, significant enrichment was observed in SEBBCH07Lik vs. SEBBCH08Lik, particularly in “carbon metabolism”, “starch and sucrose metabolism”, “glycolysis/gluconeogenesis”, and “glyoxylate and dicarboxylate metabolism”, highlighting the critical regulation of energy and carbon fluxes during early root development (**Supplementary Data Sheet S4**—Sheet B).

**Figure 7:**
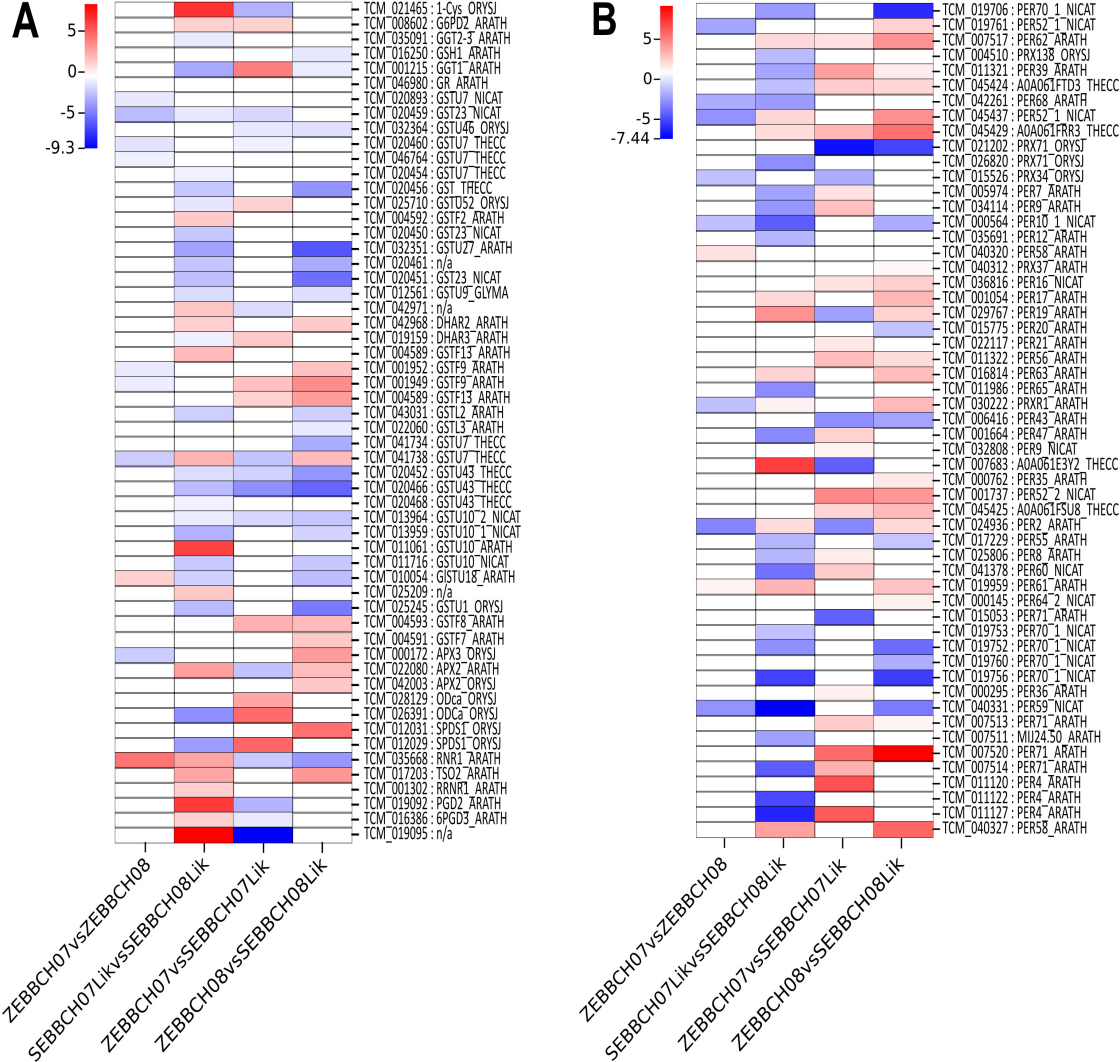
Analysis of DEGs associated to glutathione metabolism and phenylpropanoid biosynthesis pathway in the process of early cacao root development. Heatmaps of DEGs associated with **(A)** glutathione metabolism, **(B)** peroxidases in ZEBBCH07 vs. ZEBBCH08, SEBBCH07Lik vs. SEBBCH08Lik, ZEBBCH07 vs. SEBBCH07Lik, and ZEBBCH08 vs. SEBBCH08Lik pairwise comparison groups. Expression levels are indicated by the heatmap for different groups. n/a, uncharacterized gene.

An in-depth examination of phenylpropanoid biosynthesis and glutathione metabolisms revealed transcriptional modulation across experimental groups. Most notably, 55 peroxidase-related DEGs were markedly overrepresented in the phenylpropanoid pathway and 56 DEGs were mapped to glutathione metabolism in SEBBCH07Lik vs. SEBBCH08Lik comparison. Among these, 34 peroxidase-related and 39 glutathione-associated DEGs were identified, with 11 and 15 transcripts upregulated and 23 and 24 downregulated, respectively (**Figures 7A, B**; **Supplementary Figures 2D, E**; **Supplementary Data Sheet S5**—Sheet D). In parallel, the ZEBBCH07 vs. ZEBBCH08 comparison uncovered 10 and 9 DEGs in these two metabolic routes, most of which were upregulated (8/10 and 7/9). Together, these patterns point to a potentially conserved stress-responsive module activated during early root development, irrespective of the regeneration pathway. Notably, among glutathione metabolism-related DEGs, glutathione *S*-transferases (GSTs, *n*=35 DEGs) and ascorbate peroxidases (APX, *n*=3 DEGs) were predominant, as presented in **Figure 7A**. Within the SE intra-group cohort, 19 GSTs (4 up- and 15 downregulated) and a single upregulated *APX* were obtained, whereas the ZEBBCH07 vs. ZEBBCH08 group exhibited 7 GSTs (1 up- and 6 downregulated) and one downregulated *APX*. These transcripts, which encode pivotal antioxidant enzymes, were posited to orchestrate the cacao plant’s adaptive responses to ROS-induced oxidative stress during cacao root morphogenesis, especially in the context of lateral root emergence. The pronounced activation of these DEGs in fully branched SES, as compared to their zygotic counterparts, implicated a heightened antioxidative machinery in this SES line. Conversely, a poor management of oxidative stress pathways might contribute to the impaired lateral root formation observed in SES.

### 3.6 Hormonal signalling dynamics regulating root development in zygotic and somatic seedlings

**Figure 8:**
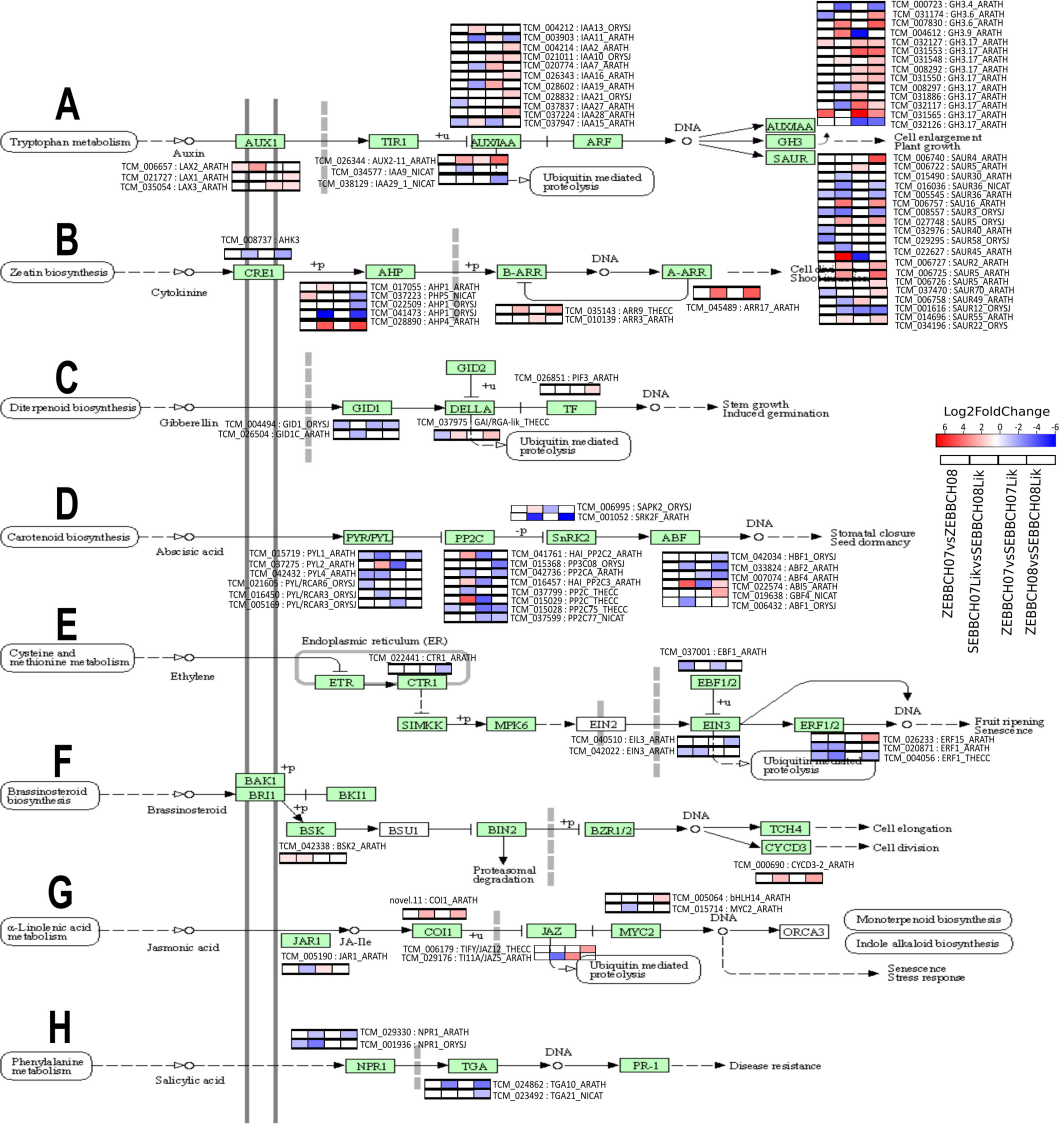
Analysis of DEGs associated to plant hormone signal transduction in the process of early cacao root development. DEG expression enriched in **(A)** auxin, **(B)** cytokinin, **(C)** gibberellin, **(D)** abscisic acid, **(E)** ethylene, **(F)** brassinosteroid, **(G)** jasmonic acid, and **(H)** salicylic acid signaling pathways (Kanehisa and Goto, 2000), in ZEBBCH07 vs. ZEBBCH08, SEBBCH07Lik vs. SEBBCH08Lik, ZEBBCH07 vs. SEBBCH07Lik, and ZEBBCH08 vs. SEBBCH08Lik pairwise comparisons. Expression levels are indicated by the heatmap for different groups. n/a, uncharacterized gene.

Based on KEGG analysis, a total of 27, 61, 38, and 71 plant hormone signal transduction-associated DEGs were significantly enriched, respectively, between ZEBBCH07 vs. ZEBBCH08, SEBBCH07Lik vs. SEBBCH08Lik, ZEBBCH07 vs. SEBBCH07Lik, and ZEBBCH08 vs. SEBBCH08Lik (**Figure 8**; **Supplementary Figure 2F**; **Supplementary Data Sheet S5**—Sheet E).

Eight distinct hormonal pathways, namely, auxin, cytokinin (CK), abscisic acid (ABA), gibberellin (GA), ethylene (ETH), brassinosteroid (BR), jasmonic acid (JA), and salicylic acid (SA) signaling pathways, were differentially modulated; the greatest count of identified DEGs was clustered within auxin and ABA pathways. Approximately 50 DEGs related to auxin transport, conjugation, and signaling were identified among all our comparison groups (**Figure 8A**). Fourteen DEGs related to amido synthetase Gretchen Hagen 3 (GH3) were enriched in auxinconjugation. Among them, 3, 6, 10, and 11 (with 2, 3, 8, and 9 upregulated and 1, 3, 2, and 2downregulated, respectively) were identified in ZEBBCH07 vs. ZEBBCH08, SEBBCH07Lik vs. SEBBCH08Lik, ZEBBCH07 vs. SEBBCH07Lik, and ZEBBCH08 vs. SEBBCH08Lik. In auxin signaling, 14 AUX/IAA and 19 SAUR DEGs were identified, which included 2 and 5 (all downregulated) in ZEBBCH07 vs. ZEBBCH08; 7 and 13 (3 and 7 upregulated and 4 and 6 downregulated, respectively) in SEBBCH07Lik vs. SEBBCH08Lik; 6 and 4 (5 and 2 upregulated and 1 and 2 downregulated) in ZEBBCH07 vs. SESBBCH07Lik; and 8 and 12 (2 and 8 upregulated and 6 and 4 downregulated, respectively) in ZEBBCH08 vs. SEBBCH08Lik, respectively. Three DEGs auxin influx carriers TCM_021727 (*LAX1_ARATH*), TCM_006657 (*LAX2_ARATH*), and TCM_035054 (*LAX3_ARATH*) were identified. *LAX2_ARATH* was differentially expressed in ZEBBCH07 vs. ZEBBCH08 and SEBBCH07Lik vs. SEBBCH08Lik while *LAX1_ARATH* and *LAX3_ARATH* were exclusively found in ZES vs. SES comparisons. These LAX genes were all upregulated in the different pairwise comparison groups. Overall, auxin-related DEGs, particularly those involved in transport (*LAX* genes) and conjugation (*GH3* family), were predominantly upregulated in ZES. Surprisingly, auxin biosynthetic genes *TAR2_ARATH* (TCM_012202), *YUC3_ARATH*, *YUC8_ARATH*, and *YUC10_NICAT* (TCM_020470, TCM_003800, and TCM_004531), and *FMO1_1_NICAT* (TCM_046237, TCM_046238, and TCM_046235, TCM_006473) were upregulated in lateral-rootless SES compared to normal root SES, with Log2FoldChanges ranging from 2.71 to 8 (**Supplementary Table 2**). Likewise, *TAR2_ARATH*, *FMO1_NICAT* (TCM_046237), and*YUC8_ARATH* genes were upregulated in lateral-rootless SES compared to ZEBBCH07seedlings. In the ZEBBCH07 vs. ZEBBCH08 comparison, *YUC3_ARATH* (TCM_020470) and*FMO1_1_NICAT* (TCM_046237 and TCM_006473) genes were highly expressed in ZEBBCH07 seedlings (**Supplementary Table 2**).

Within the CK signaling circuit, nine DEGs were identified, including one homolog of *AtHK3*, five putative histidine phosphotransfer (AHPs), two B-type response regulator (B-type RR) genes, and one A-type RR gene (**Figure 8B**). CK signaling components displayed nuanced expression patterns, with certain RRs (TCM_045489: *ARR17_ARATH* and TCM_035143: *ARR3_THECC*) upregulated in SEBBCH07Lik vs. SEBBCH08Lik, potentially reflecting altered cell division dynamics. In the GA signaling pathway, it was observed that GID1 genes were downregulated in all the comparison groups whereas a DELLA (*GAI/RGA-like_THECC*) was downregulated in ZEBBCH07 vs. ZEBBCH08 and upregulated in SEBBCH07Lik vs. SEBBCH08Lik (**Figure 8C**). The signaling pathways of ABA, ETH, BR, JA, and SA have been enriched with 22, 7, 2, 6, and 4 DEGs, respectively. The core of identified DEGs involved in ABA circuit featured downregulation of PYL/RCAR receptors, PP2C phosphatases, SRK2E kinase, and SAPK2 and bZIP TFs (ABI5/ABF), except for divergent patterns in SEBBCH07Lik vs. SEBBCH08Lik (**Figure 8D**). DEGs involved in the ETH signaling pathway including a CTR1 gene, two EIN/EI3, three *ERF* genes, and an *EBF* gene were downregulated (**Figure 8E**). A component of the BR signaling pathway, a *BSK2* gene was upregulated in ZEBBCH07 vs. ZEBBCH08 and SEBBCH07Lik vs. SEBBCH08Lik (**Figure 8F**). Concerning the JA signaling pathway, DEGs were not found in ZEBBCH07 vs. ZEBBCH08, whereas JAR1, JAZ, and MYC2 genes were downregulated in the SEBBCH07Lik vs. SEBBCH08Lik group (**Figure 8G**). In the SA transduction pathway, identified DEGs encode NPR1 and TGA proteins, which were generally downregulated across all the comparison groups (**Figure 8H**). The clustering of hormone-related DEGs into distinct expression modules further highlighted the developmental and physiological divergence between ZES and SES, with auxin-related genes and ABA genes mostly enriched (**Supplementary Figure 4A**).

### 3.7 MAPK signalling in early cacao roots as an integrative regulator of stress and developmental pathways

**Figure 9:**
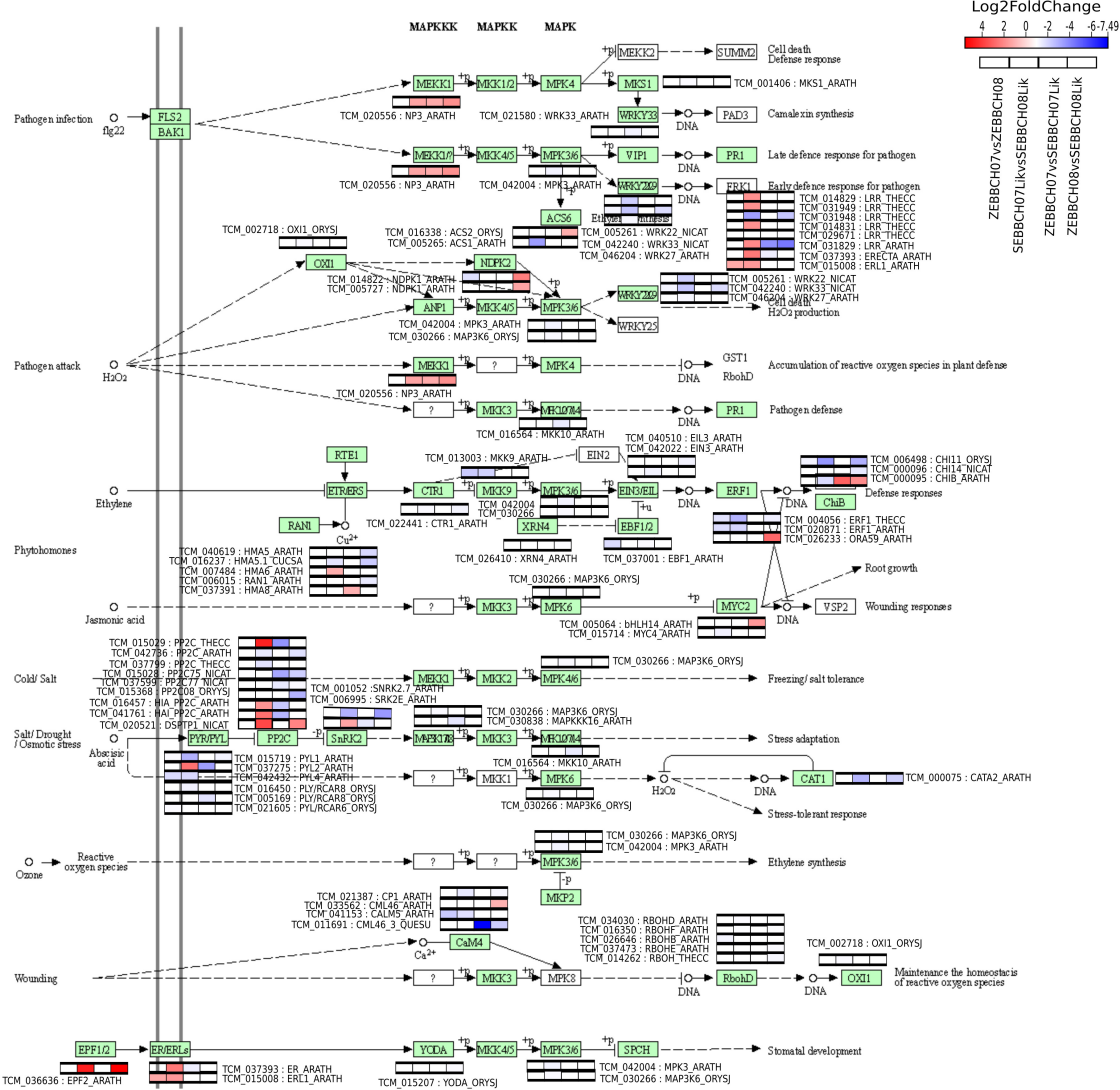
DEGs associated with the MAPK signalling pathway during early cacao root development. Specific biological processes regulated by MAPK signalling include plant hormone responses, stomatal development, pathogen defence, osmotic stress, wounding, and both hydrogen peroxide (H₂O₂) and broader ROS related signalling (Kanehisa and Goto, 2000).” These processes are represented across the ZEBBCH07 vs. ZEBBCH08, SEBBCH07Lik vs. SEBBCH08Lik, ZEBBCH07 vs. SEBBCH07Lik, and ZEBBCH08 vs. ZEBBCH08Lik pairwise comparisons. Expression patterns for each subgroup are shown in the heatmap. n/a: uncharacterized gene.

MAPK cascades function as core signaling modules downstream of sensors and receptors, coordinating cellular responses to stress adaptation, growth, and development in plants (Xu and Zhang, 2015). In the present study, KEGG analysis revealed that the MAPK signaling pathway was significantly enriched in the SEBBCH07Lik vs. SEBBCH08Lik comparison (Supplementary S4). Within this pathway, 74 DEGs were identified, primarily associated with plant hormone signaling, oxidative stress responses—including hydrogen peroxide (H_2_O_2_) and ROS metabolism—wounding, and pathogen infection (**Figure 9**; **Supplementary Figure 2F**; **Supplementary Data Sheet S5**—Sheet G). Hormonal signaling centered on ETH, JA, and ABA. Within the ETH signaling circuit, 20 DEGs were identified; notably, *MKK9* was downregulated in both ZES and SES intra-group comparisons, while *MPK3/6* was specifically downregulated in SEBBCH07Lik vs. SEBBCH08Lik. Additionally, three chitinase genes (CHI; TCM_000095, TCM_006498, and TCM_000096)—homologous to *CHI1B_ARATH*, *CHI14_NICAT*, and *CHI11_ORYSJ*—were downregulated in the SEBBCH07Lik vs. SEBBCH08Lik group. Two *ACS* DEGs (TCM_005265 and TCM_016338) involved in ETH biosynthesis were downregulated in SEBBCH07Lik vs. SEBBCH08Lik but upregulated in ZEBBCH08 vs. SEBBCH08Lik. Regarding ABA and JA signaling, *MKKK16_ARATH* and *MKK10_ARATH* were downregulated in both SEBBCH07Lik vs. SEBBCH08Lik and ZES/SES comparisons, while the JA-related transducer *MYC4_ARATH* was downregulated in the SEBBCH07Lik vs. SEBBCH08Lik comparison. Oxidative stress analysis identified 10 DEGs enriched in the H_2_O_2_ pathway. In the SEBBCH07Lik vs. SEBBCH08Lik group, *MEEK1* was upregulated, whereas the catalase gene CAT2_ARATH and several regulators of H_2_O_2_ reduction—including WRKY TFs (*WRKY22*, *27*, and *33*), *MKK10*, and *MPK3/6*—were downregulated. Furthermore, key regulators of ROS homeostasis during wounding, such as calmodulin (CaM), respiratory burst oxidase homologs (RbohD), and OXI1, were enriched in the SES intra-comparison. Specifically, *CP1*, three *CMLs*, five *RBOHs*, and *OXI1* were downregulated in SEBBCH07Lik vs. SEBBCH08Lik (**Figure 9**). Heatmap clustering demonstrated distinct expression profiles between ZES and SES, as well as between lateral-rootless and normal-root SES (**Supplementary Figure 4B**). Notably, the ZEBBCH07 vs. ZEBBCH08 comparison yielded fewer DEGs (19 DEGs; 18 downregulated) than the SEBBCH07Lik vs. SEBBCH08Lik comparison (46 DEGs; 17 upregulated and 29 downregulated).

### 3.8 Transcriptional signatures of homologous recombination and epigenetic regulation of cacao early root development

Our data report significantly enriched homologous recombination pathway exclusively in the ZEBBCH07 vs. ZEBBCH08 comparison (**Supplementary Data Sheet S2**). DEGs involved in this pathway are presented in **Figure 5C** and **Supplementary Figure 2C**, **Supplementary Data Sheet S5**—Sheet H, including replication protein A 70 kDa DNA-binding subunit D (*RPA70D_ARATH*), DNA repair protein RAD50 homolog 2 (*RAD50_ARATH*), DNA repair protein RAD51 homolog 2 (*RAD51_ARATH*), BREAST CANCER SUSCEPTIBILITY 1 homolog (*BRCA1_ARATH*) and 2 homolog B (*BRC2B _ARATH*), Double-strand break repair protein MRE11 (*MRE11_ARATH*), and crossover junction endonuclease MUS81 (*MUS81_ARATH*). All these DEGs were upregulated except for a *BRISC/BRCA1-A_ARATH* ortholog gene, TCM_037260. Meanwhile between SEBBCH07Lik vs. SEBBCH08Lik, 11 DEGs were found to be associated to the homologous recombination pathway, including DNA repair protein XRCC3 homolog (*XRCC3_ARATH*), DNA polymerase delta small subunit (*DPOD2_ARATH*), and BRCA1-associated RING domain protein 1 (*BARD1_ARATH*). Except for *RAD50_ARATH* and *BRISC/BRCA1-A_ARATH* genes, these DEGs were upregulated (**Figure 5C**). Collectively, the enrichment of homologous recombination pathway DEGs indicates active mitosis in early roots. The upregulation of canonical DNA−repair DEGs (e.g., *RAD51*, *BARD1*, *XRCC3*, *MRE11*, and *MUS81*) in ZES relative to SES suggests a stronger capacity for error−free double−strand break repair in ZES, potentially safeguarding genomic integrity during mitotic expansion—an ability that appears comparatively weaker in SES. Consistent with this pattern, histone methylation and acetylation−related DEGs (*SETs* and *GNATs*) also showed higher expression in SES than in ZES and were elevated in well−developed SES compared to lateral−rootless ones (**Table 1**; **Supplementary Data Sheet S6**). *SETs* and *GNATs* emerged as critical for regulating gene expression and cellular physiology through epigenetic gene silencing or activation. Our results suggest that coordinated shifts in histone methylation and acetylation may contribute to the regulatory landscape underlying root morphogenesis. This balance likely supports transcriptional plasticity necessary to overcome *in vitro* stress and reprogram cells for organ initiation and differentiation.

**Table 1:** Distribution of transcription factors (TFs) from the most enriched TF families identified during the process of early cacao root development. Expression changes of up- and downregulated TFs across pairwise comparisons (left panel), and counts of up- and downregulated TFs per family in each comparison (right panel).

	Log2FoldChange								Number of TFs per family								
	Up-regulated				Down-regulated				Up-regulated				Down-regulated				
TF families	ZEBBCH07 vs. ZEBBCH08	SEBBCH07Lik vs. SEBBCH08Lik	ZEBBCH07 vs. SEBBCH07Lik	ZEBBCH08 vs. SEBBCH08Lik	ZEBBCH07 vs. ZEBBCH08	SEBBCH07Lik vs. SEBBCH08Lik	ZEBBCH07 vs. SEBBCH07Lik	ZEBBCH08 vs. SEBBCH08Lik	ZEBBCH07 vs. ZEBBCH08	SEBBCH07Lik vs. SEBBCH08Lik	ZEBBCH07 vs. SEBBCH07Lik	ZEBBCH08 vs. SEBBCH08Lik	ZEBBCH07 vs. ZEBBCH08	SEBBCH07Lik vs. SEBBCH08Lik	ZEBBCH07 vs. SEBBCH07Lik	ZEBBCH08 vs. SEBBCH08Lik	Total per family
AP2/ERF	0.00	2.67	4.18	3.09	−2.68	−3.14	−2.39	−2.66	0	19	7	44	35	26	19	15	165
MYB	2.44	2.32	2.06	2.69	−2.03	−2.39	−1.97	−2.42	5	17	17	22	14	26	9	35	145
C2H2	1.51	1.93	1.34	1.97	−1.62	−2.22	−1.72	−1.90	1	24	3	34	16	20	11	23	132
bHLH	1.30	2.51	2.58	2.35	−1.53	−2.37	−3.32	−2.13	4	15	13	18	2	23	7	16	98
NAC	1.57	2.06	2.92	2.36	−1.65	−2.35	−2.66	−3.11	3	7	6	11	7	25	12	18	89
HB	1.81	3.42	1.91	3.45	−1.27	−2.25	−1.76	−2.03	2	17	8	18	1	12	3	12	73
WRKY	1.35	1.82	1.33	2.34	−1.77	−1.81	−1.82	−1.76	3	5	3	5	12	19	7	13	67
B3	1.79	4.40	1.86	1.62	−2.07	−2.04	−4.70	−2.55	3	17	1	4	6	8	14	12	65
GRAS	0.00	1.77	2.03	1.51	−1.54	−1.71	−2.51	−2.01	0	6	1	7	10	8	6	7	45
MADS	1.26	4.27	3.45	3.04	0.00	−3.69	−3.85	−2.39	2	6	4	2	0	10	7	12	43
bZIP	0.00	3.90	1.50	1.91	−1.80	−2.41	−3.36	−2.49	0	6	2	8	4	9	5	6	40
LOB	1.01	2.44	1.14	2.84	−1.91	−2.47	−3.39	−2.14	1	8	1	9	8	5	4	3	39
SNF2	1.23	1.64	0.00	0.00	0.00	0.00	−1.39	−1.52	10	6	0	0	0	0	5	16	37
GARP	2.59	2.17	0.00	2.01	−1.84	−1.81	−1.97	−2.35	1	10	0	2	3	5	6	7	34
NF−Y	0.00	5.99	2.35	1.45	−1.94	−2.53	−3.58	−3.07	0	5	2	3	2	5	9	7	33
C3H	1.09	2.76	2.53	3.32	−1.82	−1.55	−3.87	−1.85	2	5	4	3	3	3	4	5	29
AUX/IAA	0.00	1.71	1.74	2.07	−1.24	−1.75	−1.68	−1.75	0	4	5	7	2	6	1	3	28
HSF	2.78	3.16	1.27	2.53	−1.58	−1.46	−2.21	−1.58	1	3	2	3	3	7	3	5	27
SET	1.46	1.17	0.00	1.10	0.00	−2.57	−1.87	−1.67	4	3	0	1	0	1	4	13	26
zf−HD	0.00	4.18	1.86	3.52	−1.74	−1.83	−3.77	−2.16	0	5	3	7	2	3	4	2	26
PHD	1.97	0.00	1.25	1.78	0.00	−1.08	0.00	−1.47	2	0	1	2	0	3	0	15	23
Trihelix	1.14	2.50	0.00	1.99	0.00	−1.34	−1.98	−1.58	1	6	0	3	0	4	4	4	22
GNAT	0.00	1.62	0.00	1.39	−1.24	−1.98	−1.13	−1.91	0	3	0	3	2	7	2	4	21
Others	1.38	2.31	1.37	2.82	−1.21	−1.68	−1.90	−1.96	2	7	1	8	1	7	3	8	37

### 3.9 Expression patterns of transcription factors during root development in ZES and SES

This section summarizes all TFs identified as differentially expressed across the experimental comparisons. In total, 208, 520, 278, and 588 TFs from 60 families were regulated in the ZEBBCH07 vs. ZEBBCH08, SEBBCH07Lik vs. SEBBCH08Lik, ZEBBCH07 vs. SEBBCH07Lik, and ZEBBCH08 vs. SEBBCH08Lik comparisons, respectively (**Supplementary Data Sheet S6**). These TFs include the ones already mentioned in previous sections. The distribution of the most enriched differentially expressed TF families along pairwise comparison is shown in **Table 1**. AP2/ERFs, MYBs, C2H2, bHLHs, NACs, and HBs were the most represented TF families with 165, 145, 132, 98, 89, and 73 members, respectively. Notably, many TFs that regulate gene networks driving lateral root formation were identified, including AP2/ERF-EFR, AUX/IAA, bHLH, LOB, MADS-box, B3-ARF, C2C2-GATA, MYB, NAC, and WRKY. ZES intra-comparison showed fewer changes and more stable expression patterns, whereas SES, especially those forming lateral roots, featured a higher total number and greater intensity of TFs compared to ZES.

**Figure 10:**
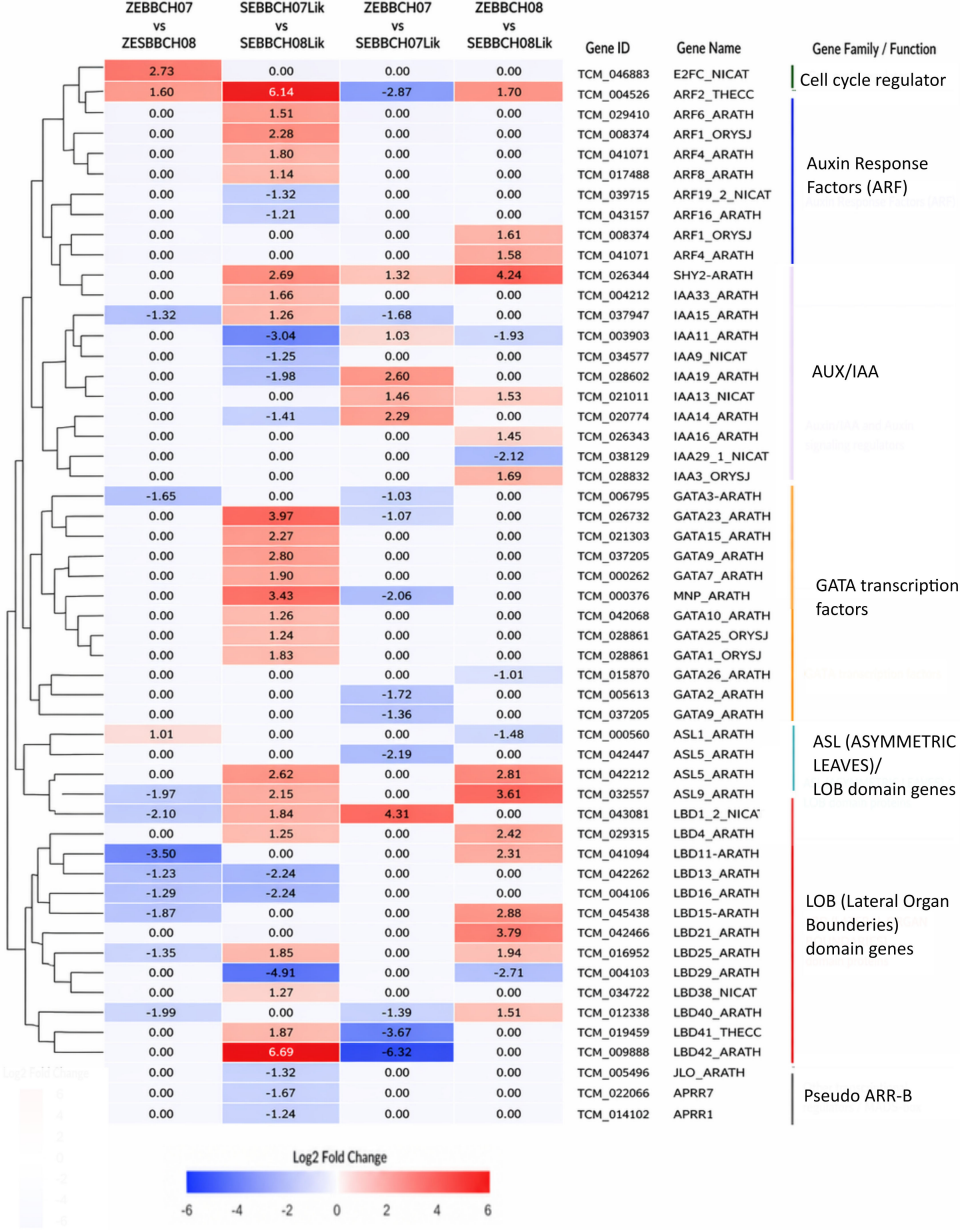
Differential expression of lateral root regulatory genes in cacao zygotic and somatic seedlings. Heatmap representation of Log2FoldChange (log_2_FC) values illustrating the differential expression of key regulatory genes involved in lateral root development across four pairwise comparisons: ZEBBCH07 vs. ZESBBCH08, SEBBCH07Lik vs. SEBBCH08Lik, ZEBBCH07 vs. SEBBCH07Lik, and ZEBBCH08 vs. SEBBCH08Lik. Each row corresponds to a gene ortholog, and each column represents a biological comparison between zygotic embryo-derived seedlings (ZES) and somatic embryo-derived seedlings (SES). Color scaling indicates relative expression changes, with red representing upregulation and blue indicating downregulation.

Cacao orthologs of key regulators of lateral root development in well-characterized systems were examined. In ZES intra-group comparison, TCM_046883*:E2FC_NICAT* and TCM_000560*:ASL1_ARATH* genes were upregulated in ZEBBCH07 seedlings while TCM_037947*:IAA15_ARATH* and several LOB domain family genes including TCM_042262*:LBD13_ARATH*, TCM_004106*:LBD16_ARATH*, and TCM_004103*:LBD29_ARATH* were upregulated in ZEBBCH08 seedlings (**Figure 10**). In lateral-rootless SES, genes related to lateral root initiation including TCM_026344*:SHY2-ARATH* and TCM_006795*:GATA3* were found to be upregulated. Auxin signaling genes TCM_037947*:IAA15_ARATH* and TCM_004212*:IAA33_ARATH* were also upregulated. Meanwhile, well developed SES showed upregulation of auxin responsive genes (TCM_020774: IAA14/*SLR1_ARATH*; TCM_028602: *IAA19_ARATH*; TCM_034577: *IAA9_NICAT*; TCM_039715: *ARF19_2_NICAT*; and TCM_043157: *ARF16_ARATH*), LOB domain family genes (TCM_042262: *LBD13_ARATH*; TCM_004106: *LBD16_ARATH*; TCM_004103: *LBD29_ARATH*; and TCM_005496: *JLO_ARATH*), and pseudoresponse regulators (TCM_014102: *APRR1* and TCM_022066: *APRR7*) (**Figure 10**; **Supplementary Data S6—Sheet C**).

## 4 Discussion

SE is a key regeneration system in plant biotechnology that enables clonal propagation and facilitates genetic improvement through stable genetic transformation and genome editing. It represents fertile ground for propagating elite and stress-resilient cacao genotypes, bypassing the limitations inherent to sexual reproduction. However, the commercial and research scalability of cacao SE is severely constrained by pervasive root system defects during the embryo-to-seedling conversion stage. Despite its importance, the molecular mechanisms governing cacao root development—and the specific failures occurring during SE—remain poorly understood. To address this knowledge gap, we compared the anatomical root structures and growth patterns of cacao ZES and SES and complemented these analyses with comparative transcriptomics to uncover the gene regulatory networks underpinning root development. Rather than serving as strict age-matched controls, ZES (BBCH07/08) were utilized as a biological benchmark for successful root architecture, providing a standardized model of productive development against which “normal” (BBCH08Lik) and “arrested” (BBCH07Lik) SES morphotypes were evaluated.

The transcriptional patterns observed in SES are consistent with chronic *in vitro* culture stress that may contribute to the structural abnormalities in stele architecture observed histologically. Comparative morphogenesis between ZES and SES during early establishment revealed clear divergences in root system plasticity and developmental synchrony. ZES followed a coordinated developmental trajectory, with radicle elongation producing a primary root of approximately 1 cm by 4 DAI (BBCH07; **Figure 1D**), followed by the emergence of lateral roots by 7 DAI (BBCH08; **Figure 1E**). As expected, lateral roots originate from founder cells derived from pericycle cells located at the xylem poles (Banda et al., 2019). Histological analyses of ZES confirmed a well-organized root architecture, with clearly defined epidermal, cortical, endodermal, and pericycle layers. The central vascular cylinder exhibited a hexarch stele, a polyarch configuration that developmentally expands the pericycle domain and increases the number of potential sites for lateral root initiation. This organization reflects advanced vascular differentiation that supports efficient transport of signaling molecules and nutrients required for sustained root growth (Hunziker and Greb, 2024). In contrast, SES exhibited pronounced morphological heterogeneity, with many seedlings displaying rootless or poorly branched phenotypes and reduced vigor. Such variability is consistent with reports across species showing that SE frequently induces developmental asynchrony and aberrant organogenesis, particularly affecting root system formation (Corredoira et al., 2019; Desai et al., 2022; Mitrofanova et al., 2022). Based on root architecture, SES could be classified into three morphotypes: (i) rootless, (ii) lateral−rootless (bearing only a primary taproot), and (iii) fully branched seedlings with primary, secondary, and tertiary roots. SES with well−developed root systems showed normal growth and complete root architecture, whereas rootless or lateral−rootless seedlings exhibited clear growth deficiencies, underscoring the central role of lateral roots in overall seedling vigor (Guan et al., 2016). Histologically, impaired SES roots displayed incomplete tissue organization, with misaligned pericycle cells and marked disruptions in vascular patterning. Their vascular cylinders contained irregularly distributed vessel strands, and several vessel elements appeared partially elongated or misaligned. Such vascular mispatterning likely compromises the identity or proliferative competence of pericycle cells, thereby preventing lateral root initiation as previously reported in *Arabidopsis* woodenleg and maize mutants (Scheres et al., 1995; Landoni et al., 2000).

These anatomical defects are strongly supported by the transcriptomic signatures uncovered in this study. ZES, which displayed orderly tissue organization and a stele organization conducive to lateral root initiation, also showed coordinated upregulation of genes involved in vascular patterning, pericycle activation, and early lateral root specification. Motor proteins, including kinesins, myosins, dyneins, and tubulins, exhibited distinct expression patterns correlating with developmental outcomes. Their higher expression in ZES supported their role in facilitating intracellular transport, cell division, and expansion necessary for root morphogenesis (Reddy, 2001; Lee et al., 2015; Nebenführ and Dixit, 2018). Conversely, the upregulation of microtubule depolymerizing (TCM_037890: *Kin13B_MAIZE*) in lateral root-deficient SES may disrupt cortical microtubule arrays, which are critical for guiding cell expansion and vascular strand alignment (Moores and Milligan, 2006; Hamant et al., 2008; Walczak et al., 2013). Disruption of microtubule organization can directly affect xylem strand continuity and alignment, thereby contributing to the vascular mispatterning observed histologically. Furthermore, the upregulation of *TcXTH* genes in ZES suggested enhanced cell wall plasticity, facilitating cell expansion and root elongation (Rose et al., 2002; Baumann et al., 2007; Cosgrove, 2018). The downregulation of these genes in SES, particularly in the lateral root-deficient type, may contribute to their impaired root system architecture.

Coupled with the anatomical evidence, this study revealed dynamic shifts in plant hormone signaling across our seedling types with auxin and ABA pathways prominently enriched (**Figure 8A**; **Supplementary Figure 2F**), reflecting their intertwined roles in plant growth and stress tolerance during SE. Auxin, the primary regulator of root branching, requires both local biosynthesis and directional transport to establish maxima in xylem pole pericycle cells (Lavenus et al., 2013; Laskowski and Tusscher, 2017). Predominantly, auxin polar transport (*LAX* family) genes were upregulated in ZES compared with SES (**Figure 8A**; **Supplementary Table 2**), suggesting that a more effective auxin transport network in zygotic embryos supports the formation of local auxin maxima, potentially explaining their enhanced root development capacity. When comparing SEBBCH07Lik vs. SEBBCH08Lik, auxin biosynthetic genes (including *TAR2* and seven *FMO/YUC* members), as well as *LAX* and *GH3* family genes, were significantly upregulated in lateral-rootless SES (SEBBCH07Lik). This concurrent elevation of biosynthesis (*TAR*/*YUC*) and conjugation (*GH3*) suggests a profound disruption of auxin homeostasis. While this could reflect a compensatory feedback mechanism in response to impaired auxin distribution, it likely points to a constitutive disruption of homeostasis that impairs downstream signaling or tissue responsiveness (Wang et al., 2023). This latter model is supported by findings in *Arabidopsis*, where gain-of-function shy2 mutants accumulate elevated endogenous IAA despite displaying a rootless phenotype (Goh et al., 2012). These transcriptomic signatures suggest that in cacao SES, developmental arrest is driven by a failure in auxin perception or local distribution rather than a production deficiency. As previously demonstrated through LC-MS metabolite profiling in cacao (Noah et al., 2021), direct quantification of endogenous auxin levels in these specific morphotypes will be a crucial future step to clarify whether this gene expression pattern results in localized auxin sequestration or systemic over-accumulation. Extending this reasoning, the downregulation of core ABA signaling components—including PYR/PYL receptors, PP2C and SnRK2 transducers, and ABF TFs—during the ZEBBCH07-to-ZEBBCH08 transition mirrors the physiological decline in ABA levels that characterizes the shift from germination to active seedling growth (Ali et al., 2022). In contrast, the more heterogeneous expression profile observed during the SEBBCH07Lik-to-SEBBCH08Lik transition (**Figure 8D**), coupled with an overall heightened ABA signaling signature in SES, may antagonize auxin-mediated pathways and suppress lateral root initiation (Zhang et al., 2023).

Beyond ABA, additional hormone−signaling pathways including CK, GA, ETH, and JA were differentially modulated across developmental stages and seedling types. Notably, lateral−rootless SES exhibited impaired CK and GA signaling (**Figures 8B, C**). CK response regulators were strongly upregulated, suggesting a shift in auxin–CK crosstalk toward enhanced CK activity at the expense of auxin−driven root proliferation. GA signaling was also perturbed. GID1 receptor genes were downregulated across all comparisons, while DELLA (*GAI_THECC*) expression decreased in ZEBBCH07 vs. ZEBBCH08 but increased in SEBBCH07Lik vs. SEBBCH08Lik. The elevated DELLA levels in lateral-rootless SES align with the well-documented growth-inhibitory effects of DELLA proteins (Achard et al., 2008). Moreover, Hetherington et al. (2021) demonstrated that exogenous GA can rescue short lateral−root phenotypes in *Arabidopsis*, supporting the inference that lateral−rootless SES likely experience reduced bioactive GA levels. Classically, ETH modulates root growth through interactions with auxin and ABA, whereas JA activates antioxidant defences and broader stress-response networks (Liu et al., 2014; Ali et al., 2025). Consistent with these roles, fully lateral-branched SES showed upregulation of ETH- and JA-responsive genes, indicative of robust stress-adaptive regulatory feedback that supports proper root system formation and resilience (Tsukagoshi, 2016). Together, these hormone−signalling shifts likely influence cell wall dynamics, defence regulation, and developmental plasticity, collectively shaping the divergent root phenotypes observed among SES morphotypes.

More broadly, this study highlighted the MAPK signaling pathway as an integrative hub for developmental and stress-responsive processes during cacao root morphogenesis, especially under the challenging conditions of SE. MAPK-associated DEGs spanned key functions in stress-responsive hormone signaling (ETH, ABA, and JA), oxidative stress response, wounding, and pathogen defence. The predominant downregulation of *MKK9*, *MPK3/6*, *ACS*, and *MYC2* genes suggests a dampened hormonal stress response in lateral-rootless SES. Additionally, several DEGs involved in ROS homeostasis including WRKY TFs, catalases, and RbohD were also downregulated in lateral-rootless SES, suggesting compromised detoxification of ROS and increased oxidative damage, reported detrimental to root meristem activity and lateral root initiation (Danquah et al., 2014; Xu and Zhang, 2015). Otherwise, the upregulation of *MEEK1* and receptor-like kinases, as well as certain innate immunity genes in these seedlings, pointed to a compensatory activation of defence and patterning pathways (**Figure 9A**). However, this response likely appeared insufficient to restore normal root architecture in the absence of robust MAPK-ROS regulatory feedback. Simultaneously, analysis of phenylpropanoid biosynthesis and glutathione metabolism (**Supplementary Figures 2D, E**; **Supplementary Data S5- Sheets E, F**) revealed pronounced transcriptional modulation of antioxidant enzymes, notably peroxidases, glutathione-*S*-transferases, and ascorbate peroxidases. The heightened expression of these antioxidative genes in fully branched SES compared to their zygotic counterparts suggests an enhanced protective mechanism against oxidative damage incurring during *in vitro* culture. Conversely, misregulation of these pathways in lateral root-deficient SES likely contributes to developmental impairment by allowing ROS accumulation to disrupt regulatory networks critical for lateral root organogenesis (Manzano et al., 2014; Tsukagoshi, 2016). Interestingly, a significant upregulation of beta-alanine, taurine, and hypotaurine metabolic pathways in SEBBCH07Lik seedlings suggested that they are likely experiencing higher abiotic stress compared to the fully branched SES. Furthermore, hypotaurine has been implicated in the inhibition of lateral root formation (Fang et al., 2014). Thus, these amino acids may serve as biomarkers of impaired root system development in cacao.

Additionally, the strong upregulation of nitrogen, amino acid, carbon, and carbohydrate metabolism in SEBBCH08Lik seedlings (**Figure 6A**; **Supplementary Data Sheet S4**–Sheet B) likely reflects increased metabolic demand to sustain root growth rather than a response solely to *in vitro* induced stress (Kawade et al., 2023; Teixeira et al., 2020). Pathways such as starch and sucrose metabolism, glycolysis, and the glyoxylate cycle were particularly elevated in SES with lateral roots, suggesting enhanced carbon flux for energy production. The previously reported activation of the glyoxylate cycle in cacao somatic embryos (Noah et al., 2013) further supports its function as a stress-adaptive mechanism that mobilizes stored lipids to fuel root development under *in vitro* conditions. Together, these observations indicate that metabolic reprogramming under *in vitro* conditions reflects a growth–defense trade−off that ultimately shapes root development.

In conjunction with these metabolic shifts, we hypothesize that histone methyltransferase (*SETs*) upregulation in ZEBBCH07 (early lateral root primordia stage) likely enforces H3K9/K27 methylation to silence proliferative genes, stabilizing cell fate during initial root patterning and preventing premature differentiation. Acetyltransferase (*GNATs*) upregulation in ZEBBCH08 (lateral root emergence) may promote H3/H4 acetylation, activating auxin-responsive genes like *PLETHORA* (*PLT*) or *LATERAL ROOT PRIMORDIUM 1* (*LRP1*) for meristem maintenance and lateral branching, enabling progression from primordia to outgrowth (Tersenidis et al., 2024). The elevated expression of both SET and GNAT histone modifiers in SES suggests that these seedlings may undergo substantial epigenetic reprogramming under *in vitro* stress, consistent with transcriptional patterns previously associated with methylome instability during cacao SE (Garcia et al., 2022). Well-developed SES showed higher SET/GNAT expression than lateral-rootless morphotypes, suggesting that increased histone-modifying capacity may correlate with the transcriptional activation of root-branching programs and with the repression of stress-associated genes. These patterns also point to a broader epigenetic distinction between ZES and SES: ZES exhibit a more uniform transcriptional profile consistent with coordinated developmental progression, whereas SES display more variable and elevated expression of epigenetic regulators, potentially reflecting greater transcriptional plasticity under *in vitro* conditions. Although functional consequences cannot be inferred solely from transcript abundance, these findings raise the hypothesis that modulating SET and GNAT activity could improve SES conversion efficiency in cacao and direct chromatin-level assays (e.g., ChIP-seq or ATAC-seq) would be suitable to validate the functional impact of these transcriptional shifts on cacao root morphogenesis. Similarly, upregulation of homologous recombination genes—including *RAD50*, *RAD51*, *MRE11*, and *MUS81* in ZES—may reflect a robust DNA repair system safeguarding genomic integrity during rapid cell proliferation (Oh and Symington, 2018; Banerjee and Roy, 2021). In contrast, the downregulation of *MRE11* and *MEI1* in SES points to a putative deficiency in the double-strand break repair machinery. Whether this transcriptional signature translates into measurable genomic instability remains a hypothesis for future investigation through direct DNA damage quantification assays.

Interestingly, the upregulation of TCM_046883*:E2FC_NICAT* and TCM_000560*:ASL1_ARATH* genes in ZEBBCH07 (**Figure 10**) suggests a potential link with the reactivation of the cell cycle in xylem pole pericycle cells and the establishment of founder cell identity. Similarly, the induction of TCM_037947*:IAA15_ARATH* and several *LOB* genes, including TCM_042262*:LBD13_ARATH*, TCM_004106*:LBD16_ARATH*, and TCM_004103*:LBD29_ARATH* in ZEBBCH08, likely reflects the progression toward auxin-driven primordium outgrowth (Lavenus et al., 2013; Banda et al., 2019). Likewise, well-developed SES displayed induction of genes associated with sustained auxin signaling, primordium patterning, and developmental progression, including *IAA14/19_ARATH*, *ARF16_ARATH*, *ARF19_2_NICAT*, *LBD13/16/29_ARATH*, *JLO_ARATH*, and *APRR1/7_ARATH*. This reflects a complete activation of the lateral root regulatory network. In contrast, lateral-rootless SES exhibited strong upregulation of SHY2/IAA3 and GATA23 alongside the absence of LBD16/29 induction, a pattern consistent with the modular organization of auxin signaling during lateral root formation. Within this framework, Aux/IAA–ARF modules act sequentially to regulate founder cell specification, primordium initiation, and subsequent emergence. The induction of GATA23 indicates activation of the IAA28–ARF7/19 module responsible for specifying lateral root founder cells, suggesting that initial priming responses remain functional in these SES. By contrast, the lack of LBD16 and LBD29 expression points to a failure to engage the downstream SLR/IAA14–ARF7/19 module required for asymmetric cell divisions and primordium initiation. This differs from well-developed SES, where robust LBD16/29 expression reflects successful progression into organogenic development. The concurrent upregulation of SHY2/IAA3 in lateral-rootless SES provides a plausible mechanistic basis for this developmental arrest; as a negative regulator of auxin signaling, SHY2/IAA3 restricts the ARF activity necessary for sustained organogenesis. Consistent with Goh et al. (2012), heightened SHY2/IAA3 activity can lead to a decoupling between early transcriptional activation (founder cell specification) and the actual morphogenetic output (primordium emergence), effectively stalling the developmental program.

## 5 Conclusion

Our findings provided compelling evidence that the root formation deficiencies observed in SES resulted from a convergence of disruptions in both morphogenetic cell patterning and transcriptional regulation. Jointly, the interplay between epigenetic and primary metabolic processes, MAPK signaling, hormonal regulation, and antioxidant and DNA damage responses underscored a complex adaptive network essential for successful root development and stress tolerance in cacao. These insights advanced our understanding of the physiological and molecular mechanisms governing root growth and lateral branching in ZES and in normal and misshaped SES. They opened avenues for targeted genetic or biotechnological interventions to enhance root morphogenesis and stress adaptation efficiency, seedling vigor, and field establishment of elite cacao genotypes and other woody crops. Potential biomarkers of SES production improvement emerging from this comparative study with ZES may include the upregulation of *MKK9*, *MPK3/6*, *ACS*, *MYC2*, catalases, and *RbohD* genes, as well as genes related to auxin (e.g., *LBD16/29*), ABA and GA signaling, cytoskeleton motor proteins, and homologous recombination genes such as *RAD50*, *RAD51*, *MRE11*, and *MUS81*. Future investigations could consider refining the chemical composition of culture media to mitigate the detrimental effects on post-embryonic development, therefore enhancing the regeneration rate of SE. Ultimately, these results provide a valuable foundation for optimizing breeding strategies, contributing to the sustainable improvement of cacao productivity and resilience in the face of environmental challenges.

## Data Availability

The authors confirm that all relevant data supporting the findings of this study are available within the article and its supplementary material. BioProject and associated SRA metadata are available at: NCBI, PRJNA1457099.

## Supplementary material

Supplementary Figure 1 Bar chart of DEGs counts in the top 10 GO terms belonging to “MF”, “CC” and “BP” categories.

Supplementary Figure 2 Relative gene expression levels of DEGs involved in the key KEGG pathways during the process of early cacao root development in ZEBBCH07, ZEBBCH08, SEBBCH07Lik and SEBBCH08Lik root seedlings.

Supplementary Figure 3 Gene co-expression clusters analysis of zygotic and somatic seedlings during the process of early cacao root development.

Supplementary Figure 4 Heatmaps of DEG associated with plant hormone signal transduction and MAPK signalling pathways in the process of early cacao root development.

Supplementary Table 1 RNA sample quantification, integrity and purity.

Supplementary Table 2 DEGs related to auxin biosynthetic pathway in the process of early cacao root development.

Supplementary Data Sheet 1 RNA sequencing analysis and screening of DEGs between pairs of samples.

Supplementary Data Sheet 2 List of DEGs in intra-ZES/SES and cross pairwise comparison groups.

Supplementary Data Sheet 3 GO terms significantly enriched across pairwise comparisons.

Supplementary Data Sheet 4 KEGG pathways identified across pairwise comparisons.

Supplementary Data Sheet 5 Counts, Log2FoldChanges and relative gene expression level values of DEGs involved in the key KEGG pathways in the process of early cacao root development.

Supplementary Data Sheet 6 Distribution of all transcription factors (TFs) identified during the process of early cacao root development.
