# Supplementary material for: Transcriptional regulation of root development in cacao: a genome-wide comparative analysis between zygotic and somatic embryo-derived seedlings
Source: Front Plant Sci. 2026 Jun 3;17:1816111. doi: 10.3389/fpls.2026.1816111 (PMC13272332; doi:10.3389/fpls.2026.1816111)
Supplement: Supplementary Figure 1 — Bar chart of DEGs counts in the top 10 GO terms belonging to “MF”, “CC” and “BP” categories. [file Table1.docx]

**Supplementary Figure S1:** Bar chart of DEGs counts in the top 10 GO terms belonging to “MF”, “CC” and “BP” categories. Numbers of up-/down-regulated DEGs in (A) ZEBBCH07 vs. ZEBBCH08, (B) SEBBCH07Lik vs. SEBBCH08l, (C) ZEBBCH07 vs. SEBBCH07Lik, (D) ZEBBCH08 vs. SEBBCH08l pairwise comparisons. In the figure, the horizontal axis is the number of DEGs, the vertical axis is the GO term, and the bar colour represents three categories in the Gene Ontology database. BP = “biological process”, CC = “cellular component” and MF = “molecular functions”. GO terms highlighted using “*” are significantly enriched.

**Supplementary Figure S2:** Relative gene expression levels of DEGs involved in the key KEGG pathways during the process of early cacao root development in ZEBBCH07, ZEBBCH08, SEBBCH07Lik and SEBBCH08l root seedlings. A- Motor proteins; B- Cell remodeling; C- Homologous recombination; D- Glutathione metabolism; E- Phenylpropanoid biosynthesis pathway; F- Plant hormone signaling; G-MAPK signalling.

**Supplementary Table S1:** RNA sample quantification, integrity and purity.

**Supplementary Table S2:** DEGs related to auxin biosynthetic pathway in the process of early cacao root development.

**Supplementary Data S1:** RNA sequencing analysis and screening of DEGs between pairs of samples

**Supplementary Data S2:** List of DEGs in intra-ZES/SES and cross pairwise comparison groups. Sheet A- Details of DEGs presented in clustered heatmap of DEGs across all root samples (Figure 3C). Sheet B- Detailed list of genes expressed in all root samples (ZEBBCH07, ZEBBCH08, SEBBCH07Lik and SEBBCH08l). Detailed list of up-/down-regulated DESs found in (C) ZEBBCH07 vs. ZEBBCH08, (D) SEBBCH07Lik vs. SEBBCH08l, (E) ZEBBCH07 vs. SEBBCH07Lik, (F) ZEBBCH07 vs. SEBBCH08l pairwise comparisons.

**Supplementary Data S3:** GO terms significantly enriched across pairwise comparisons. Sheet (A) ZEBBCH07 vs. ZEBBCH08, (B) SEBBCH07Lik vs. SEBBCH08l, (C) ZEBBCH07 vs. SEBBCH07Lik, (D) ZEBBCH07 vs. SEBBCH08l pairwise comparisons.

**Supplementary Data S4:** KEGG pathways identified across pairwise comparisons. Sheet (A) ZEBBCH07 vs. ZEBBCH08, (B) SEBBCH07Lik vs. SEBBCH08l, (C) ZEBBCH07 vs. SEBBCH07Lik, (D) ZEBBCH07 vs. SEBBCH08l pairwise comparison groups. KEGG pathways highlighted in red are significantly enriched.

**Supplementary Data S5:** Counts, Log2FoldChanges and relative gene expression level values of DEGs involved in the key KEGG pathways in the process of early cacao root development. Sheet A- Motor protein and cell wall remodelling pathways; Sheet B- Amino acid and nitrogen metabolism; Sheet C- Carbon and carbohydrate metabolism; Sheet D- Glutathione metabolism; Sheet E- Phenylpropanoid biosynthesis pathway; Sheet F- Plant hormone signaling; Sheet G- MAPK signalling plant; Sheet H- Homologous recombination pathway.

**Supplementary Data S6:** Distribution of all transcription factors (TFs) identified during the process of early cacao root development. Sheet A- Expression changes of up- and down-regulated TFs per family across pairwise comparisons (left panel) and counts of up- and down-regulated TFs per family in each comparison (right panel). Sheet B- Detailed list of up- and down-regulated TFs across pairwise comparisons.

**Supplementary Figure S1**

**
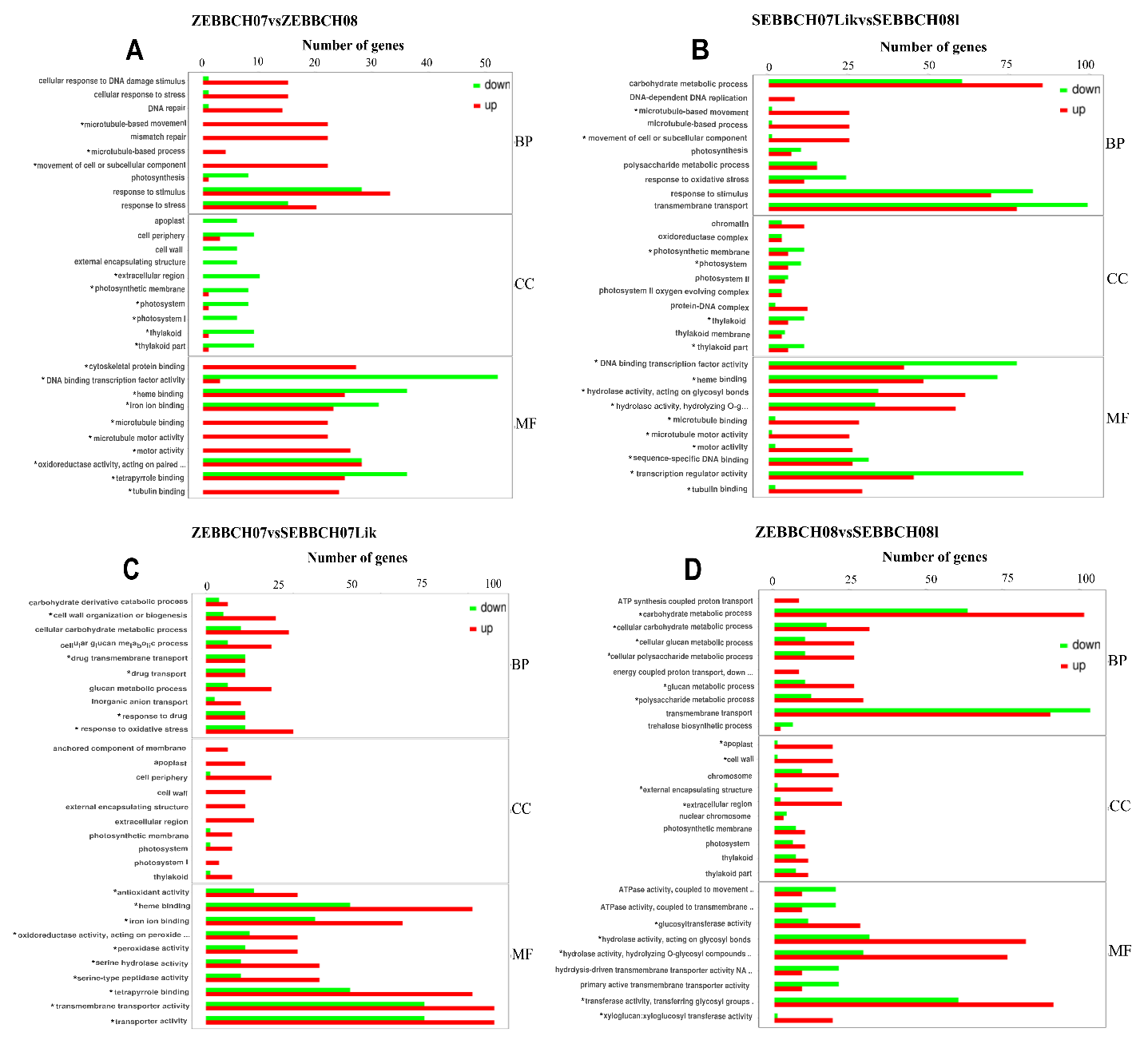
**

**Supplementary Figure S2**

**Supplementary Table S1:** RNA sample quantification, integrity and purity.

| **Sample Name** | **Concentra-tion (ng/ul)** | **Volume (ul)** | **Total amount (ug)** | **Integrity value** | **DO 260/280** | **DO 260/230** | **Sample QC Results** |
| --- | --- | --- | --- | --- | --- | --- | --- |
| ZEBBCH071 | 466.27 | 8 | 3.73 | 9.8 | 2.30 | 2.53 | Pass, Smooth baseline |
| ZEBBCH072 | 80.05 | 8 | 0.64 | 9.1 | 2.04 | 1.65 | Pass, Smooth baseline |
| ZEBBCH073 | 188.16 | 9 | 1.69 | 9.8 | 2.15 | 2.01 | Pass, Smooth baseline |
| ZEBBCH081 | 558.15 | 9 | 5.02 | 9.8 | 2.13 | 2.09 | Pass, Smooth baseline |
| ZEBBCH082 | 348.59 | 9 | 3.14 | 9.7 | 2.17 | 2.28 | Pass, Smooth baseline |
| ZEBBCH083 | 169.68 | 9 | 1.53 | 9.8 | 2.18 | 2.17 | Pass, Smooth baseline |
| SEBBCH07Lik1 | 266.13 | 6 | 1.6 | 9.3 | 2.25 | 2.29 | Pass, Smooth baseline |
| SEBBCH07Lik2 | 27.96 | 7 | 0.2 | 9.4 | 2.22 | 1.94 | Pass, Smooth baseline |
| SEBBCH07Lik3 | 31.14 | 8 | 0.25 | 8.8 | 2.13 | 2.00 | Pass, Smooth baseline |
| SEBBCH08l1 | 305.13 | 8 | 2.44 | 9.7 | 2.14 | 2.24 | Pass, Smooth baseline |
| SEBBCH08l2 | 145.64 | 7 | 1.02 | 9.8 | 2.19 | 1.65 | Pass, Smooth baseline |
| SEBBCH08L3 | 158.26 | 9 | 1.42 | 9.8 | 2.1 | 2.05 | Pass, Smooth baseline |

The RNA samples demonstrated high quality and suitability for sequencing applications, with concentrations ranging from 27.96 to 558.15 ng/µL and total amounts between 0.2 and 5.02 µg. Integrity values consistently exceeded 8.8, indicating excellent RNA integrity RNA and hence intact and high-quality. Purity ratios (A260/280 and A260/230) were close to or above 2.0, reflecting minimal protein and organic compound contamination.

**Supplementary Table S2**: DEGs related to auxin biosynthetic pathway in the process of early cacao root development.

| **Gene ID** | **Gene symbols** | **Log2FoldChange** | | | |
| --- | --- | --- | --- | --- | --- |
|  |  | **ZEBBCH07 vs. ZEBBCH08** | **SEBBCH07Lik vs. SEBBCH08l** | **ZEBBCH07 vs. SEBBCH07Lik** | **ZEBBCH08 vs.**  **SEBBCH08l** |
| TCM_012202 | TAR2_ARATH | 0* | 3,48 | -2,23 | 0 |
| TCM_046237 | FMO1_1_NICAT | 4,09 | 8,00 | -1,78 | 0 |
| TCM_046238 | FMO1_1_NICAT | 0 | 4,63 | 0 | 0 |
| TCM_038236 | FMO1_3_NICAT | 0 | -1,56 | 0 | 0 |
| TCM_037420 | FMO1_THECC | 0 | -2,97 | 0 | 0 |
| TCM_006473 | FMO1_1_NICAT | 1,10 | 2,99 | 0 | 2,04 |
| TCM_046235 | FMO1_1_NICAT | 0 | 3,58 | 0 | 0 |
| TCM_020470 | YUC3_ARATH | 2,31 | 2,72 | 0 | 0 |
| TCM_003800 | YUC8_ARATH | 0 | 5,18 | -3,02 | 0 |
| TCM_004531 | YUC10_NICAT | 0 | 2,88 | 0 | 2,40 |

*0 means no differential expression; TAR, Tryptophan aminotransferase-related protein; FMO, Putative flavin-containing monooxygenase; YUC, Probable indole-3-pyruvate monooxygenase.
