# Supplementary material for: Transcriptional regulation of root development in cacao: a genome-wide comparative analysis between zygotic and somatic embryo-derived seedlings
Source: Front Plant Sci. 2026 Jun 3;17:1816111. doi: 10.3389/fpls.2026.1816111 (PMC13272332; doi:10.3389/fpls.2026.1816111)
Supplement: Supplementary file 2 [file Table2.docx]

**Supplementary S1**

**RNA sequencing analysis**

To explore the molecular events associated with cacao root development, a high-resolution transcriptome analysis was performed on the entire root system of the four seedling types -ZEBBCH07, ZEBBCH08, SEBBCH07Lik, and SEBBCH08l - each with three biological replicates. After trimming and filtering, Illumina RNA‑seq of the 12 libraries yielded 85.6 Gb of clean data comprising 570,745,118 paired‑end reads. The average GC content was 45.44 ± 0.68% while the Q30 was comprised between 92.88 and 94.03%. An average of 92.95% of the raw reads were uniquely mapped to the *T. cacao* reference genome with mapping rates ranging from 89.45% to 94.60% (Table 2). These high mapping rates indicated that the reference genome provided suitable template for aligning reads.

To assess the uniformity of sequencing across samples and quantify the normalized gene expression level, Fragments Per Kilobase of transcript per Million mapped reads (FPKM) values were calculated for each sample. Our results showed distinct but partially overlapping gene expression patterns among samples, as indicated by non-identical peaks in the density distributions (Fig. 10A and B). Furthermore, the median FPKM values (log2(fpkm+1)) were remarkably consistent and closely aligned across groups, as evidenced by the proximity of lines in the density plots (Fig. 10B). Such results attested the high quality and uniformity of the RNA-Seq data, minimizing technical bias across samples. On the other hand, the gene expression distribution indicated a high degree of similarity among group samples’ expressional profiles (Fig. 10C), reinforcing the suitability and the reliability of our RNA-seq data.

Further, correlations and PCA analysis were performed on 12 samples from our four root tissues. Correlation analyses demonstrated strong intra-group reproducibility (Pearson’s correlation coefficient R² > 0.898; Fig. 11A) whereas, PCA analysis distinctly separated the four root-tissue morphotypes (Fig. 11B). Notably, ZEBBCH07 and ZEBBCH08 samples clustered more closely than their SES counterparts. Hierarchical clustering delineated discrete expression trends in the different root samples (Fig. 11C; Supplemental S2-Sheet-A). These results collectively substantiate the existence of morphotype-specific regulatory programs and the large-scale developmental shifts during root formation in both ZES and SES.

**Screening of DEGs between pairs of samples**

Pairwise differential expression analysis was conducted with stringent thresholds (padj < 0.05 and |log2FoldChange| ≥ 1.0) using DEseq2 software (Varet et al., 2016). A total 2,371 and 5,835 DEGs were identified in ZEBBCH07 vs ZEBBCH08 and SEBBCH07Lik vs SEBBCH08l, respectively. Of these, 1,102 and 2,973 were up-regulated, while 1,269 and 2,862 were down-regulated. Cross-comparisons between ZES and SES root systems yielded 3,409 and 6,998 DEGs in ZEBBCH07 vs SEBBCH07Lik and ZEBBCH08 vs SEBBCH08l, respectively, with up-regulated genes consistently outnumbering down-regulated ones (Table 3; Supplemental S2-Sheet-B, C, D, E, F). Notably, 607/636, 1218/1036, 692/921, and 1385/1399 up-/down-regulated DEGs were uniquely expressed in ZEBBCH07 vs ZEBBCH08, SEBBCH07Lik vs SEBBCH08l, ZEBBCH07 vs SEBBCH07Lik and ZEBBCH08 vs SEBBCH08l, respectively, while 24/21 up-/down-regulated genes were commonly regulated across these comparison groups (Fig. 11D).

Volcano plots graphically depicted the magnitude and significance of gene expression changes, revealing considerable variations in the fold changes of gene expression levels between root samples (Supplemental Fig. 10D). Our analysis indicated a broad spectrum of log2FoldChange within each pairwise comparison, with absolute values surpassing 25 and -log10(padj) up to 200, indicative of pronounced transcriptional divergence between root phenotypes.

**Table 2:** Quality statistics of original sequencing data and alignment analysis of filtered data with reference genome sequence.

| **Samples** | **Raw reads** | **Raw bases** | **Clean reads** | **Clean bases** | **Q20** | **Q30** | **% GC** | **Error rate** | **Total mapped** | **Unique mapped** |
| --- | --- | --- | --- | --- | --- | --- | --- | --- | --- | --- |
| ZEBBCH071 | 42187410 | 6.33G | 40417410 | 6.06G | 97.5 | 93.29 | 45.15 | 0.03 | 38039286 (94.12%) | 36875654 (91.24%) |
| ZEBBCH072 | 44825964 | 6.72G | 43337132 | 6.5G | 97.75 | 93.82 | 44.85 | 0.03 | 40996771 (94.6%) | 39835437 (91.92%) |
| ZEBBCH073 | 44174342 | 6.63G | 42667518 | 6.4G | 97.35 | 92.88 | 45.18 | 0.03 | 40343930 (94.55%) | 39166372 (91.79%) |
| ZEBBCH081 | 40795226 | 6.12G | 40002630 | 6.0G | 97.65 | 93.71 | 46.43 | 0.03 | 36640768 (91.6%) | 35037210 (87.59%) |
| ZEBBCH082 | 41610028 | 6.24G | 40628622 | 6.09G | 97.49 | 93.27 | 46.24 | 0.03 | 36947651 (90.94%) | 35495804 (87.37%) |
| ZEBBCH083 | 53042516 | 7.96G | 51913414 | 7.79G | 97.45 | 93.24 | 46.91 | 0.03 | 46438949 (89.45%) | 44629946 (85.97%) |
| SEBBCH07Lik1 | 65635280 | 9.85G | 63121534 | 9.47G | 97.64 | 93.7 | 44.87 | 0.03 | 58557448 (92.77%) | 56663748 (89.77%) |
| SEBBCH07Lik2 | 56513282 | 8.48G | 55126272 | 8.27G | 97.88 | 94.03 | 44.85 | 0.03 | 51668072 (93.73%) | 50482030 (91.58%) |
| SEBBCH07Lik3 | 51373608 | 7.71G | 50346006 | 7.55G | 97.83 | 93.98 | 45.23 | 0.03 | 46256339 (91.88%) | 45247599 (89.87%) |
| SEBBCH08l1 | 53069422 | 7.96G | 50618144 | 7.59G | 97.72 | 93.72 | 45.19 | 0.03 | 47789991 (94.41%) | 46544123 (91.95%) |
| SEBBCH08l2 | 44658104 | 6.7G | 42811244 | 6.42G | 97.7 | 93.74 | 45.12 | 0.03 | 40283543 (94.1%) | 39370535 (91.96%) |
| SEBBCH08l3 | 51487244 | 7.72G | 49755192 | 7.46G | 97.71 | 93.78 | 45.34 | 0.03 | 46417500 (93.29%) | 45254078 (90.95%) |

**Table 3:** The differentially expressed genes (DEGs) number among pairwise comparisons using an absolute value of |log2FoldChange|≥1.0 and adjusted p-value (padj) <0.05 as significant cutoff.

| DEG set name | All DEG counts | Up-regulated | Down-regulated |
| --- | --- | --- | --- |
| ZEBBCH07vsZEBBCH08 | 2371 | 1102 | 1269 |
| SEBBCH07LikvsSEBBCH08l | 5835 | 2973 | 2862 |
| ZEBBCH07vsSEBBCH07Lik | 3409 | 1795 | 1614 |
| ZEBBCH08vsSEBBCH08l | 6998 | 3644 | 3354 |


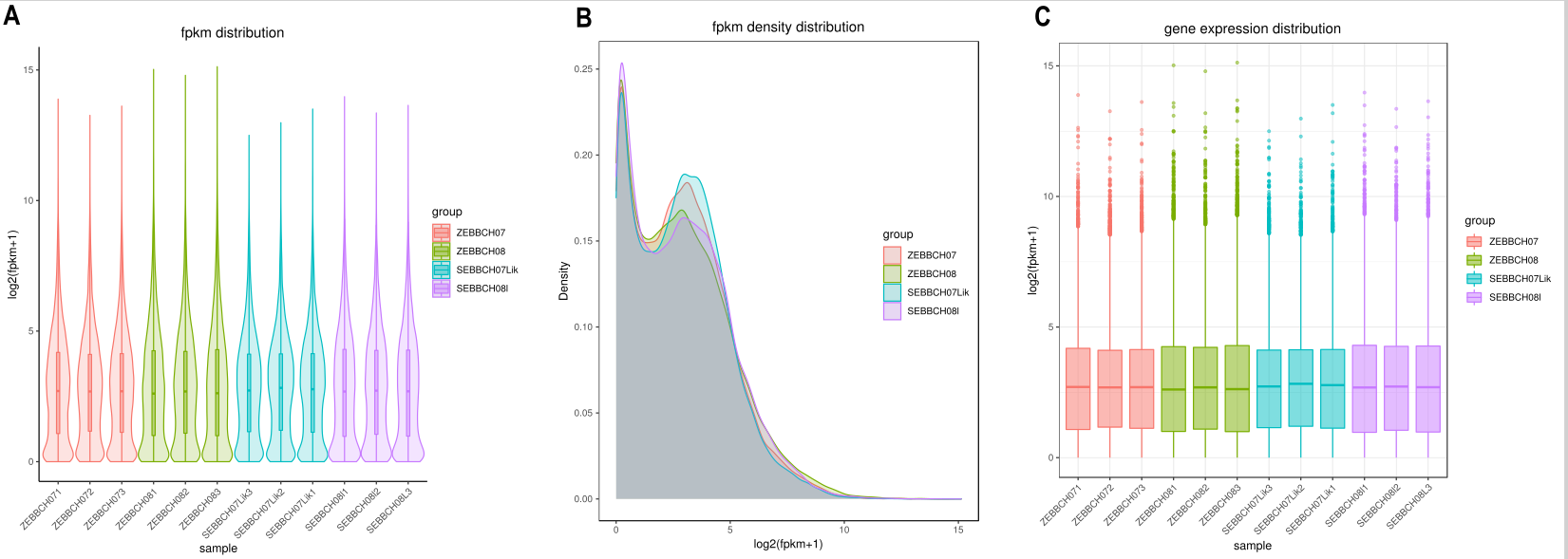


**D**

**Fig. 10:** Transcriptome data analysis of cacao root tissues from ZEBBCH07, ZEBBCH08, SEBBCH07Lik and SEBBCH08l seedlings, SCA6 genotype. A- Fragments Per Kilobase of transcript per Million mapped reads (FPKM) distribution, B- FPKM density, C- Gene expression distribution. D- Volcano plots displaying the distribution of DEGs across (a) ZEBBCH07 vsZ EBBCH08, (b) SEBBCH07Lik vs SEBBCH08l, (c) ZEBBCH07 vs SEBBCH07Lik, (d) ZEBBCH08 vs SEBBCH08l pairwise comparisons. The horizontal axis represents the log2FoldChange value, and the vertical axis represents -log10padj, the dashed line represents the threshold line for screening differential genes. Red and green represent up-regulated and down-regulated genes, respectively.


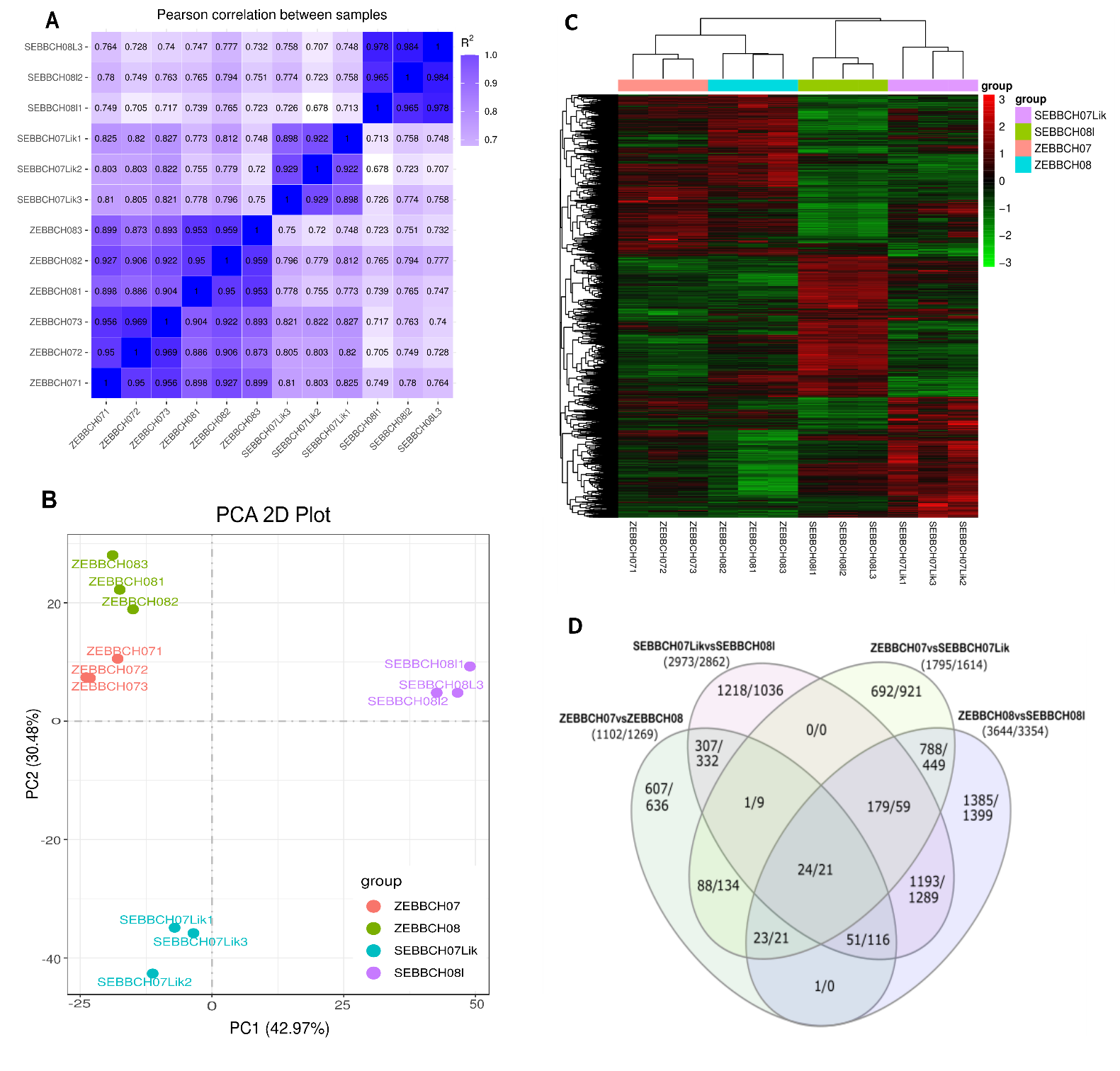


**Fig. 11:** Expression analysis of DEGs of cacao roots from ZEBBCH07, ZEBBCH08, SEBBCH07Lik and SEBBCH08l seedlings, SCA6 genotype. A- Pearson’s correlation analysis, the value of r2 ranges from 0 to 1, and the closer the r2 value is to 1, the higher the degree of correlation. B- Biplot of the first and second principal components (PC1 vs. PC2) generated from all root samples. C- Clustered heatmap of DEGs across all root samples. D- Venn diagram illustrating number of shared and unique up-/down-regulated DEGs between ZEBBCH07 vs ZEBBCH08, SEBBCH07Lik vs SEBBCH08l, ZEBBCH07 vs SEBBCH07Lik and ZEBBCH08 vs SEBBCH08l pairwise comparison groups.
